# *Helicobacter pylori* versus the Host: Remodeling of the Bacterial Outer Membrane Is Required for Survival in the Gastric Mucosa

**DOI:** 10.1371/journal.ppat.1002454

**Published:** 2011-12-22

**Authors:** Thomas W. Cullen, David K. Giles, Lindsey N. Wolf, Chantal Ecobichon, Ivo G. Boneca, M. Stephen Trent

**Affiliations:** 1 Section of Molecular Genetics and Microbiology, The University of Texas at Austin, Austin, Texas, United States of America; 2 Institut Pasteur, Group Biology and Genetics of the Bacterial Cell Wall, Paris, France; 3 INSERM, Groupe Avenir, Paris, France; 4 The Institute of Cellular and Molecular Biology, The University of Texas at Austin, Austin, Texas, United States of America; Fred Hutchinson Cancer Research Center, United States of America

## Abstract

Modification of bacterial surface structures, such as the lipid A portion of lipopolysaccharide (LPS), is used by many pathogenic bacteria to help evade the host innate immune response. *Helicobacter pylori*, a gram-negative bacterium capable of chronic colonization of the human stomach, modifies its lipid A by removal of phosphate groups from the 1- and 4′-positions of the lipid A backbone. In this study, we identify the enzyme responsible for dephosphorylation of the lipid A 4′-phosphate group in *H. pylori*, Jhp1487 (LpxF). To ascertain the role these modifications play in the pathogenesis of *H. pylori*, we created mutants in *lpxE* (1-phosphatase), *lpxF* (4′-phosphatase) and a double *lpxE/F* mutant. Analysis of lipid A isolated from *lpxE* and *lpxF* mutants revealed lipid A species with a 1 or 4′-phosphate group, respectively while the double *lpxE/F* mutant revealed a *bis*-phosphorylated lipid A. Mutants lacking *lpxE, lpxF, or lpxE/F* show a 16, 360 and 1020 fold increase in sensitivity to the cationic antimicrobial peptide polymyxin B, respectively. Moreover, a similar loss of resistance is seen against a variety of CAMPs found in the human body including LL37, β-defensin 2, and P-113. Using a fluorescent derivative of polymyxin we demonstrate that, unlike wild type bacteria, polymyxin readily associates with the *lpxE/F* mutant. Presumably, the increase in the negative charge of *H. pylori* LPS allows for binding of the peptide to the bacterial surface. Interestingly, the action of LpxE and LpxF was shown to decrease recognition of *Helicobacter* LPS by the innate immune receptor, Toll-like Receptor 4. Furthermore, *lpxE/F* mutants were unable to colonize the gastric mucosa of C57BL/6J and C57BL/6J *tlr4* -/- mice when compared to wild type *H. pylori*. Our results demonstrate that dephosphorylation of the lipid A domain of *H. pylori* LPS by LpxE and LpxF is key to its ability to colonize a mammalian host.

## Author Summary

Since its discovery in 1982 *Helicobacter pylori* has been identified as the leading cause of gastritis and peptic ulcer disease, infecting around 50% of the world's population. Infected patients are at increased risk for gastric cancers, allowing for classification of *H. pylori* as a class I carcinogen by the World Health Organization. *H. pylori* has only one well defined niche, the human stomach. Since no other reservoirs exist, a unique balance must be established during infection permitting long-term survival of both the bacterium and its human host. Here, we show that *H. pylori* modifies its primary surface component, lipopolysaccharide (LPS), making the bacterium undetectable by components of the innate immune system and highly resistant to antimicrobial compounds secreted by host cells. Mutant strains of *H. pylori* unable to modify their surface show increased sensitivity to antimicrobial peptides (∼1000 fold) and increased recognition by components of the innate immune system. *H. pylori* mutants were unable to colonize mouse models, suggesting that remodeling of LPS is essential for survival within the gastric mucosa. Understanding the adaptations used by *H. pylori* to survive and persist within the human host is key towards unraveling how this unique organism impacts gastric disease.

## Introduction

*Helicobacter pylori*, a gram-negative bacterium with only one well-defined niche, the human stomach, can persist for several years without manifestation of symptoms. However, over time serious complications may appear including peptic ulcer disease and gastric cancer, allowing for classification of *H. pylori* as a class I carcinogen by the World Health Organization [1], [2]. Similar to most gram-negative bacteria the outer membrane of *H. pylori* is composed of lipopolysaccharide (LPS), a surface exposed glycolipid.

LPS is anchored to the bacterial membrane by its hydrophobic lipid A domain. Extended from lipid A is the core oligosaccharide followed by the O-antigen creating a uniform hydrophilic surface layer that interphases with the surrounding environment. The first sugar of the core, Kdo (3-deoxy-D-*manno*-octulosonic acid), serves as a bridge to link the lipid anchor to the carbohydrate domains of LPS. The core polysaccharide is well conserved within a bacterial species; however, this is not the case with O-antigen. *H. pylori* shows great diversity in expression of its O-antigen [3], achieving a form of molecular mimicry by assembling surface polysaccharides resembling human blood group antigens, contributing to its ability to evade immune detection [4].

**Figure 1:**
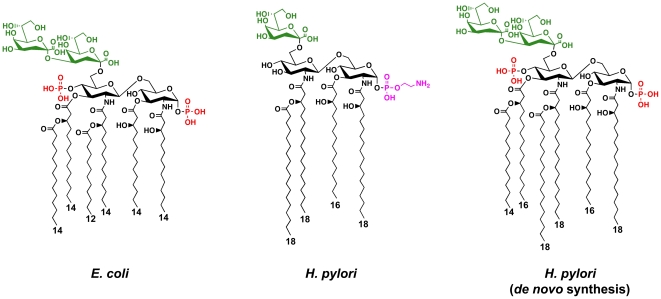
Chemical structures of the Kdo-lipid A domain of *E. coli* and *H. pylori*. The major lipid A species of *E. coli* is a hexa-acylated disaccharide of glucosamine with phosphate groups at the 1- and 4′-positions. The first sugar of the core oligosaccharide, Kdo (3-deoxy-D-*manno*-octulosonic acid), is attached at the 6′-position and serves as a bridge to link lipid A to the carbohydrate domains of LPS. The minor *de novo* lipid A species of *H. pylori* is similar to that seen in *E. coli*, but has longer acyl chains. However, the mature surface exposed major lipid A species of *H. pylori* is tetra-acylated that is lacking the 4′-phosphate group, and is substituted at the C-1 position with a phosphoethanolamine residue. Phosphate groups are shown in red, the Kdo sugars are shown in green, and the phosphoethanolamine is shown in magenta. The length of the fatty acyl chains are indicated.

The biosynthetic pathway for assembly of Kdo_2_-lipid A (Figure 1), is well conserved throughout gram-negative bacteria and proceeds via the nine-step enzymatic “Raetz pathway”; however, great variety is seen in Kdo_2_-lipid A structures when comparing gram-negative bacterial species [5]. One of the best examples of Kdo-lipid A diversity is the structure produced on the surface of *H. pylori* (Figure 1). Variation in the Kdo-lipid A domain of LPS is generated in part through the action of modification enzymes [6]. The structural diversity among human pathogens might have arisen through evolutionary pressures applied to the bacterium by the host innate immune system.

To evade the host innate immune system by modification of its lipid A, pathogenic bacteria employ several approaches. One approach involves masking of negatively charged phosphate groups present on the lipid A disaccharide backbone by adding positively charged substituents, such as phosphoethanolamine or L-4-aminoarabinose, while a second approach involves the complete removal of phosphate groups from the backbone [7]. Both approaches result in a net loss of negative surface charges, resulting in a bacterial membrane more resistant to cationic antimicrobial peptides (CAMPs). CAMPs are positively charged peptides (e.g. defensins) that bind to negatively charged surface structures (e.g. lipid A) present on the bacterial cell surface, presumably creating pores in the membrane, resulting in cell lysis and eventual death [8], [9]. These structurally diverse CAMPs are found in macrophages, neutrophils and at the mucosal surface, making them an important component of the host innate immune response [8]. It has also been reported that changes in lipid A acylation or removal of Kdo (3-deoxy-D-manno-octulosonic acid) core sugars, through the activity of modification machinery, play a role in resistance to CAMPs [10], [11].

The lipid A domain is responsible for the endotoxic properties associated with LPS due to recognition and activation of the human Toll-like receptor 4-myeloid differentiation factor 2 (hTLR4-MD2) complex [12]. The human Toll-like receptor 2 (hTLR2), known to recognize several conserved bacterial structures including lipoteichoic acid and lipoproteins, has also been shown to recognize atypical forms of LPS [13], [14]. During infection by gram-negative organisms, dissociated LPS is recognized by the hTLR4-MD2 complex present on many cell types [15]. In preventing dissemination of infection, LPS serves as a molecular signal helping to clear the invading microbe; however, over-stimulation of inflammatory mediators (e.g. TNF-α), can result in the syndrome of septic shock [16]. LPS from enteric bacteria such as *Escherichia coli* produce the typical highly stimulatory lipid A (Figure 1), easily recognized by hTLR4-MD2; however, many pathogenic bacteria produce altered lipid A structures resulting in attenuated activation of hTLR4-MD2, allowing for immune evasion [6]. Several tactics are employed to produce hTLR4-MD2 attenuated lipid A structures, including the incorporation of longer acyl chains (16 or 18 carbons) instead of the standard (12 or 14 carbons) or by decreasing the overall number of acyl-chains from hexa-acylated to penta- or tetra-acylated lipid A forms [16]. In some organisms, the masking or removal of phosphate groups on the disaccharide backbone serves a dual role in CAMP resistance and attenuation of hTLR4-MD2 activation [17].

*H. pylori* produce a minimal Kdo-lipid A structure that is tetra-acylated with a phosphoethanolamine residue attached at the 1 position of the disaccharide (Figure 1), presumably contributing to high CAMP resistance and attenuated hTLR4-MD2 activation reported for this pathogen [18]. Since no other reservoirs exist for *H. pylori*, a unique balance must be established during infection in order to permit long-term survival of both the bacterium and its human host. Unlike a number of bacterial pathogens [6], modification of *H. pylori* lipid A appears to be constitutive and is not regulated by specific environmental cues [19]. Furthermore, the *H. pylori* lipid A modification pathway is highly ordered and efficient giving rise to a single lipid A species. This is in contrast to the lipid A variation seen on the surface of other pathogens [19]. Given that only one known reservoir exists for *H. pylori*, the human stomach, adaptation to changing environmental conditions is presumably unnecessary and explains this lack of regulation and single form of lipid A.

**Figure 2:**
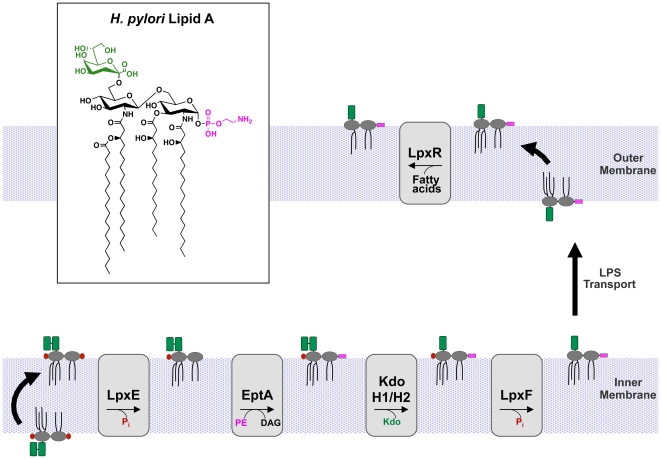
Modification pathway of *H. pylori* Kdo-lipid A. *H. pylori* produce a highly modified lipid A species via a five step enzymatic pathway. First, the 1-phosphate group is cleaved by LpxE (Jhp0019) followed by the addition of phosphoethanolamine by EptA (Jhp0020) at the 1-position. Phosphatidylethanolamine (PE) likely serves as the donor for EptA resulting in the formation of diacylglycerol (DAG). Next KdoH1 (Jhp0526) and KdoH2 (Jhp0527) act in concert to remove the terminal Kdo sugar. The 4′-phosphate group is removed by LpxF (Jhp1487) leaving an unmodified hydroxyl group, followed by removal of the 3′-*O*-linked acyl chains by LpxR (Jhp0634) resulting in a tetra-acylated lipid A. Following transport of core-lipid A across the inner membrane, the first four modification reactions occur at the periplasmic face of the inner membrane. The final step, catalyzed by LpxR, occurs in the outer membrane to produce *H. pylori* Kdo-lipid A (inset). Cartoon structures are shown to illustrate the intermediate structures in the pathway. The phosphate groups are shown in red, Kdo shown in green, and phosphoethanolamine in magenta. The core-oligosaccharide and O-antigen are not shown for simplicity.

*De novo* lipid A synthesized by *H. pylori* is modified from a *bis*-phosphorylated hexa-acylated Kdo_2_-lipid A structure (Figure 1) by a five-step enzymatic pathway (Figure 2) and a single distinct lipid A species is produced [19], [20], [21], [22]. Previous studies from our laboratory have characterized the enzymes responsible for four of these steps; however, the identity of the lipid A 4′-phosphatase in *H. pylori* has yet to be identified or characterized. Thus, what role the 4′-phosphatase plays in CAMP resistance or in modulation of hTLR4-MD2 activation has not been addressed. Furthermore, although the lipid A 1-phosphatase (LpxE) of *H. pylori* was shown to be important for CAMP resistance, its role in hTLR4-MD2 activation remains unclear [17]. Here we report the identification of the lipid A 4′-phosphatase in *H. pylori* and demonstrate that the 1 and 4′-phosphatases act synergistically to produce a bacterial surface that is highly resistant to CAMP attack. We also show that dephosphorylation of *H. pylori* lipid A at the 1 and/or 4′ position results in LPS with attenuated hTLR4-MD2 activation. Most exciting, our results suggest that modification of *H. pylori* lipid A phosphate groups are important for colonization of a host.

## Results

### The *H. pylori* Lipid A 4′-Phosphatase Is Encoded by *jhp1487*

The published structure for wild type *H. pylori* is Kdo-lipid A that is tetra-acylated with a phosphoethanolamine attached at the 1-position (Figure 1) [18]. Previous work in our laboratory identified and characterized the Kdo_2_-lipid A modification machinery in *H. pylori*, including LpxE (1-phosphatase), EptA (phosphoethanolamine transferase), KdoH1/2 (Kdo Hydrolase) and LpxR (3′-*O*-deacylase), which all act in an ordered efficient manner to produce a single lipid A species found on the bacterial surface (Figure 2) [19], [20], [22]. To date, all enzymes have been identified and characterized except for the enzyme responsible for removal of the lipid A 4′-phosphate group, also known as LpxF (4′-phosphatase).

Previous attempts to identify *H. pylori lpxF* by our laboratory have proven unsuccessful, as heterologous expression of possible homologs in *E. coli* have failed to demonstrate LpxF activity in whole cells [17]. Searching the *H. pylori* genome for proteins homologous to LpxE (Hp0021) using the Blastp algorithm [23] revealed three possibilities, identified as belonging to a family of phosphatidic acid type 2 phosphatases (PAP2), Jhp0324, Jhp0787 and Jhp1487. Along with LpxE these proteins are members of COG0671, a cluster of putative orthologs of *E. coli* PgpB. PgpB is a membrane-bound phosphatidylglycerol phosphatase involved in phospholipid biosynthesis. A ClustalW2 alignment of Jhp0324, Jhp0787 and Jhp1487 against LpxE revealed a score of 8%, 7% and 20%, respectively. Score is defined as percent identities divided by number of residues compared. Given that Jhp1487 showed the highest score (20%) when aligned with LpxE, it became the likely target. A knockout in *jhp1487* was created by interruption of the coding sequence with an antibiotic resistance cassette in *H. pylori* strain J99 and named J99 *lpxF*. Complementation of J99 *lpxF* was achieved by re-introduction of *lpxF* into the chromosome and named J99 *lpxF, lpxF^+^*.

**Figure 3:**
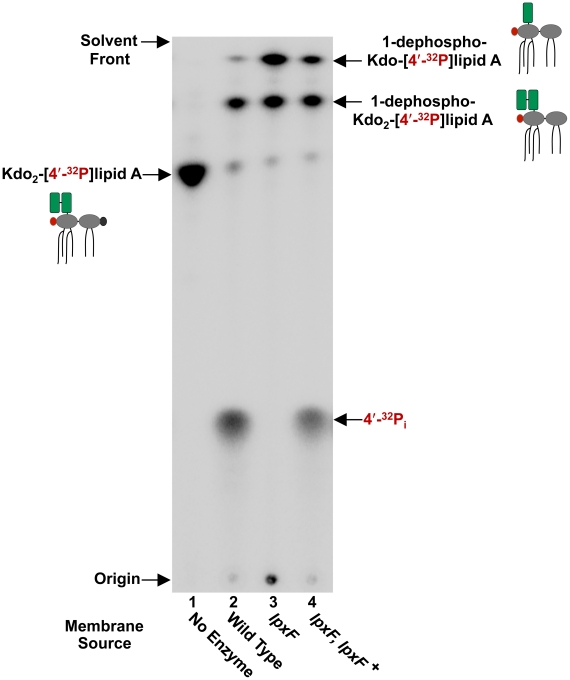
*In vitro* assay of the *H. pylori* lipid A 4′-phosphatase, LpxF. Membranes from an *lpxF* deficient mutant in strain J99 were assayed using Kdo_2_-[4′-^32^P]lipid A as the substrate. LpxF activity is indicated by the appearance of free radiolabeled inorganic phosphate (^32^P_i_). The LpxF mutant showed a complete loss of 4′-phosphatase activity, which was fully restored in the complemented strain. Reaction products from the previously characterized 1-phosphatase (LpxE) [20] and Kdo hydrolase (KdoH1/H2) [19], [21] were also detected. Cartoon structures are shown to illustrate reaction products. The 1-phosphate group is shown in black, the radiolabeled 4′-phosphate group in red, and the Kdo sugars are shown in green.

To screen for LpxF activity in membrane fractions, *in vitro* assays were performed using radiolabelled Kdo_2_-[4′^32^P]lipid A as the substrate (Figure 3). Following incubation of the enzyme source with the radioactive substrate, the reaction products were separated via thin-layer chromatography (TLC) such that the more hydrophobic reaction products migrated faster. As expected, wild type *H. pylori* J99 membranes show robust LpxF activity (lane 2) as indicated by the appearance of free inorganic phosphate (^32^P_i_), while membranes isolated from J99 *lpxF* were completely free of 4′-phosphatase activity (lane 3). LpxF activity was restored in membranes isolated from the complemented strain J99 *lpxF, lpxF^+^* (lane 4), indicating that the *H. pylori* lipid A 4′-phosphatase is encoded by *jhp1487*. Reaction products from the previously characterized 1-phosphatase (LpxE) [20] and Kdo hydrolase (KdoH1/H2) [19], [21] were also detected. Loss of *jhp1487* had no effect on 1-phosphatase or Kdo hydrolase activity. It was demonstrated previously [17] that membranes lacking a functional LpxE were unable to catalyze removal of the 1-phosphate group of lipid A substrates. Thus, ruling out *H. pylori* LpxF as a lipid A 1-phosphatase.

### Characterization of Lipid A from Select *H. pylori* Mutants

To clearly ascertain the role LpxE and LpxF play in the pathogenesis of *H. pylori*, a series of mutants were generated, including a single *lpxE, lpxF* and a double *lpxE/F* mutant. Single *lpxE* and double *lpxE/F* mutants were created by introduction of a previously described *lpxE* mutation [17] into wild type J99 and J99 *lpxF* background by natural transformation, creating J99 *lpxE* and J99 *lpxE/F*, respectively. Complementation of J99 *lpxE* was achieved by re-introduction of *lpxE* into the chromosome and named J99 *lpxE, lpxE^+^*. Additionally, all mutations were moved into the mouse adapted *H. pylori* strains, B128 and X47 [24], [25], [26].

**Figure 4:**
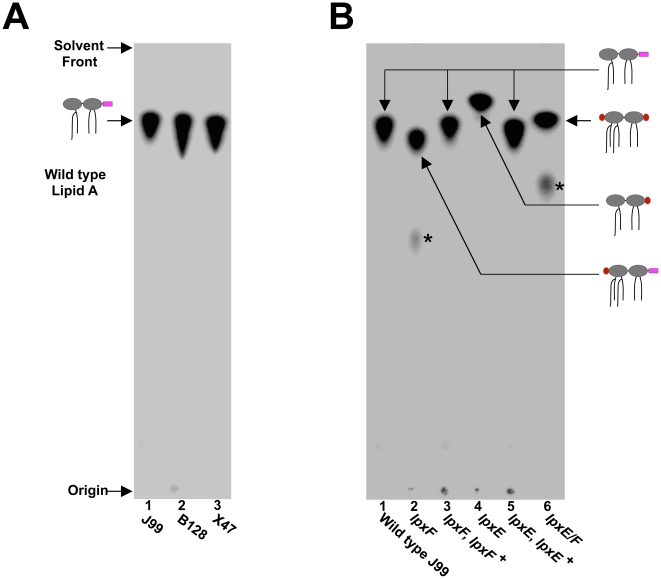
TLC analysis of ^32^P-labelled lipid A from select *H. pylori* strains. *H. pylori* were grown in the presence of ^32^P_i_, the lipid A purified, separated by TLC and visualized by phosphorimaging. (A) All strains of wild type *H. pylori* (J99, B128, and X47) revealed identical lipid A species. (B) TLC analysis of wild type J99 and complemented strains revealed a single lipid A species with migration typical of wild type *H. pylori* lipid A. Analysis of the *lpxE*, *lpxF*, and double *lpxE/F* mutants in a J99 background reveal changes in the migration pattern, indicating changes in the lipid A structure. The structure of each lipid A species was later identified by MALDI-TOF mass spectrometry analysis and cartoon structures are included for clarification. Phosphate groups are shown in red and phosphoethanolamine is shown in magenta. A minor penta-acylated lipid A species indicated by an asterisk was present in *lpxF* deficient backgrounds.

To thoroughly characterize this series of mutants, the lipid A species produced by each strain was determined. To begin, all strains were grown in the presence of ^32^P_i_, the lipid A purified, separated by TLC and visualized by phosphorimaging. *H. pylori* wild type strains J99, B128 and X47 all revealed a single identical species of lipid A indicating no variation between backgrounds (Figure 4A). In contrast, lipid A from the single *lpxE*, *lpxF* and double *lpxE*/F mutants showed differences in migration compared to that of wild type. Chromosomal complementation of either the *lpxE* or *lpxF* mutations resulted in production of wild type lipid A (Figure 4B).

**Figure 5:**
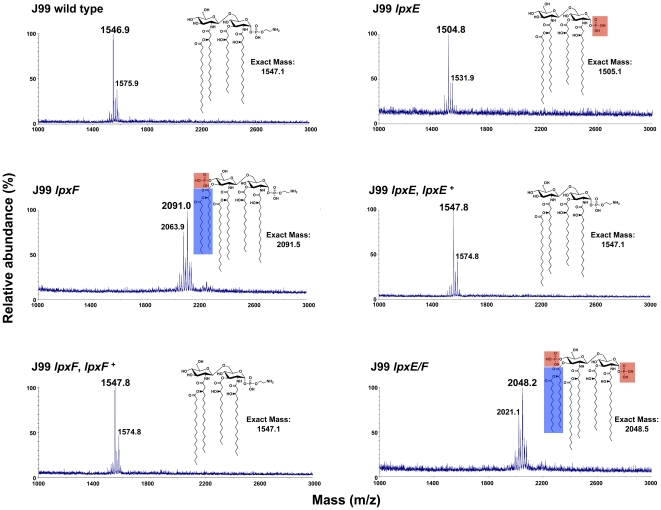
Mass spectrometry of *lpxE*, *lpxF* and *lpxE/F* mutants in strain J99. Lipid A was isolated from wild type, *lpxE*, *lpxF*, *lpxE/F* and complemented strains in strain J99 and analyzed by MALDI-TOF mass spectrometry in the negative-ion mode. Wild type and complemented stains produced a peak at 1546.9 to 1547.8 *m/z* corresponding to the published wild type *H. pylori* lipid A structure [20], [59]. The *lpxE*, *lpxF* and *lpxE/F* mutants showed a peak at *m/z* 1504.8, 2091.0 and 2048.2, respectively. 1504.8 corresponds to tetra-acylated 1-phosphorylated lipid A, 2091.0 to hexa-acylated 4′-phosphorylated lipid A and 2048.2 to hexa-acylated *bis*-phosphorylated lipid A. The lipid A structure for each strain is shown with changes to phosphate groups outlined in red and changes in acylation outlined in blue.

To confirm changes in lipid A and determine the structure, lipid A from all strains was subjected to analysis by MALDI-TOF (matrix-assisted laser desorption/ionization-time of flight) mass spectrometry in the negative ion mode. The wild type J99 spectrum showed a predominant peak at *m/z* 1546.9 consistent with the [M–H]^-^ ion of the wild type structure of *H. pylori* lipid A (predicted [M–H]^-^ at *m/z* 1547.1), which is tetra-acylated without a phosphate group at the 4′-position and a phosphoethanolamine residue at the 1-position (Figure 5). The *lpxF* mutant spectrum showed a predominant peak at *m/z* 2091.0 consistent with the [M–H]^-^ ion of a lipid A species that is hexa-acylated with a phosphate group at the 4′-position and a phosphoethanolamine residue at the 1-position (predicted [M–H]^-^ at *m/z* 2091.5), confirmation that the *H. pylori* LpxF is encoded by *jhp1487*. The presence of hexa-acylated lipid A species in the *lpxF* mutant suggest an ordered modification system in which *H. pylori* LpxR (3′-*O*-deacylase) activity is dependent on removal of the lipid A 4′-phosphate group by LpxF. The *lpxE* mutant spectrum (Figure 5) showed a predominant peak at *m/z* 1504.8 consistent with the [M–H]^-^ ion of a lipid A species that is tetra-acylated bearing a single phosphate group (predicted [M–H]^-^ at *m/z* 1505.1), in agreement with the published lipid A structure of *lpxE* deficient *H. pylori* strains [17]. The double *lpxE/F* mutant spectrum showed a predominant peak at *m/z* 2048.2 consistent with the [M–H]^-^ ion of a lipid A species that is hexa-acylated with a phosphate group at the 1- and 4′-positions (predicted [M–H]^-^ at *m/z* 2048.5) (Figure 5). Complemented *lpxE* and *lpxF* strains showed a predominate peak consistent with that of wild type *H. pylori* (Figure 5). Moreover, MALDI-TOF mass spectrometry analysis of lipid A purified from all mutants in B128 (Figure S1 in Text S1) and X47 (Figure S2 in Text S1) backgrounds, agreed with corresponding J99 stains. The proposed structure for the lipid A species found in each strain is illustrated in Figure 5.

### Dephosphorylation of Lipid A by LpxE and LpxF Results in Resistance to CAMPs

Resistance to CAMPs is of great importance to all mucosal pathogens. This is especially true for *H. pylori* whose only known reservoir is the human gastric mucosal surface. *H. pylori* is highly resistant to the CAMP polymyxin B (PMB) with the minimal inhibitory concentration (MIC) of wild type strains ranging from 250–1000 µg/ml peptide [19]. PMB is an experimental substitute for CAMPs and commonly used to demonstrate CAMP resistance in laboratory settings because of a similar mechanism of action [27]. MICs were determined for all strains using PMB Etest strips. *H. pylori* wild type strain J99 and all complemented strains exhibited a PMB MIC (469.3±73.9 µg/ml) comparable to published determinations [17], [19]. The *lpxE*, *lpxF* and double *lpxE*/*F* mutants in background J99 showed a 16, 360 and 1020 fold decrease in resistance to PMB, with MICs of 29.3±4.6, 1.3±0.3 and 0.46±0.03 µg/ml, respectively (Table 1). Thus, although LpxE and LpxF work in concert to produce a highly resistance bacterial membrane, removal of the 4′-phosphate group has the most influence on CAMP resistance in *H. pylori*.

**Table 1:** Minimal Inhibitory Concentrations (MIC) of polymyxin against *H. pylori* strains.

*H. pylori strain*	Background J99 Polymyxin MIC ( µg/ml)	Background B128 Polymyxin MIC( µg/ml)	Background X47 Polymyxin MIC( µg/ml)
*Wild Type*	469.3±73.9	213.3±37.0	768.0±0.0
*lpxF*	1.3±0.3	1.8±0.3	2.3±0.6
*lpxF, lpxF* ^+^	512.0±0.0	213.3±37.0	682.7±147.8
*lpxE*	29.3±4.6	26.7±4.6	37.3±9.2
*lpxE, lpxE* ^+^	469.3±73.9	234.7±37.0	768.0±0.0
*lpxE/F*	0.46±0.03	0.61±0.04	0.55±0.08
*lpxR*	512.0±0.0	ND	ND
*lpxR, lpxR * ^+^	469.3±73.9	ND	ND

MIC are reported as µg/ml and are the average of three experiments using Polymyxin B E-Test strips (Biomerieux) on TSA supplemented with 5% blood. ND =  Not determined.

To rule out changes in acylation in the single *lpxF* and double *lpxE*/F mutant as playing a role in CAMP resistance, the MIC of a previously characterized *lpxR* mutant was also determined and found to be comparable (512±0.0 µg/ml) to that shown by wild type *H. pylori* (Table 1). The lipid A of the *lpxR* deficient strain is hexa-acylated without a phosphate group at the 4′-position and a phosphoethanolamine residue at the 1-position [22] suggesting that, in *H. pylori*, deacylation plays no role in resistance to CAMPs. MICs were also determined for mutants in the mouse adapted backgrounds, B128 and X47, and were found to be comparable to J99 stains (Table 1).

The lifecycle and transmission of *H. pylori* is not well understood. Most literature agrees that oral familial passage of the bacterium is the most likely route of transmission, suggesting that *H. pylori* must not only resist CAMPs located at the gastric mucosal surface, but also those found in the oral cavity [28]. Moreover, the bacterium must resist CAMPs secreted by a variety of leukocytes, constantly surveying for invading pathogens [8]. To confirm our PMB MIC findings, we repeated MIC determinations using CAMPs possibly encountered by *H. pylori* during its lifecycle. We chose a number of peptides relevant to human infection for this study including: (i) the human cathelicidin LL-37 produced by both leukocytes and epithelial cells, (ii) human β-defensin 2 (HBD-2) found throughout the gastrointestinal tract, (iii) P-113, a fragment of histatin 5 found within the oral cavity, (iv) and HNP-2, an α–defensin produced by neutrophils [9], [29].

To determine the MICs, a standard microtiter broth dilution method was utilized. J99 wild type was highly resistant to all CAMPs analyzed (Table 2). The *lpxE*, *lpxF* and double *lpxE/F* mutants all showed a decrease in resistance against LL37, P-113, and HBD-2, similar to what is seen against PMB (Table 2). Once again, LpxF activity seemed to have the largest influence on resistant CAMPs. No change in resistance was seen for the α–defensin HNP2 at the indicated concentrations (Table 2). The *lpxR* deficient strain was identical to that of wild type, once again confirming that deacylation of LPS plays no role in resistance to CAMPs in *H. pylori* (Table 2). Together, these results indicate that dephosphorylation of lipid A in *H. pylori*, by LpxE and LpxF, is essential for resistance to CAMPs.

**Table 2:** Minimal Inhibitory Concentrations (MIC) of cationic antimicrobial peptides against *H. pylori* strains.

Strain	Polymyxin	HP2.20^1^	HNP2 (α-Def)^2^	HBD2 (β-Def)^3^	LL37^4^	P113^5^
J99 Wild Type	>250	>1250	>25	>25	40 ± 5	>400
J99 *lpxE*	22 ± 2	583 ± 29	>25	23 ± 3	40 ± 0	341 ± 14
J99 *lpxF*	3.8 ± 0.3	60 ± 0	>25	12 ± 1	19 ± 2	178 ± 3
J99 *lpxE/F*	0.6 ± 0.1	18 ± 3	>25	5.0 ± 1.0	11 ± 1	141 ± 14
J99 *lpxR*	>250	>1250	>25	>25	41 ± 6	>400

MIC are reported as µg/ml and are the average of three experiments.

1 *Helicobacter pylori* (2–20); ^2^ human neutrophil peptide-2 (α-defensin);

3 human beta defensin-2 (β-defensin); ^4^ human cathelicidin LL-37; ^5^ P-113 fragment of histatin-5.

Interestingly, *H. pylori* produces an antibacterial peptide referred to as Hp (2.20), related to the insect cecropins that bind to the phosphate groups of lipid A [30]. *H. pylori* is naturally resistant to Hp (2.20), and it has been suggested that release of this peptide into the stomach may contribute to clearance of other gastrointestinal pathogens providing some benefit to *H. pylori* infected individuals [30]. Again, the double *lpxE/F* mutant shows a large decrease (≥ 60 fold) in resistance against Hp (2.20) (Table 2). The level of Hp (2.20) production by *Helicobacter* during colonization of the gastric mucosa is unclear; however, it is likely that modification of the lipid A structure provides protection against this *Helicobacter* derived antimicrobial compound.

### Dephosphorylation of Lipid A by LpxE and LpxF Produce a Surface More Resistant to CAMP Binding

CAMPs primary mode of action against bacteria is to bind negatively charged moieties present on the bacterial surface (e.g. phosphate groups) [8]. Unlike most gram-negative bacteria, wild type strains of *H. pylori* completely lack unsubstituted phosphate moieties on its LPS [18]. However, LpxE/F deficient *H. pylori* strains produce an LPS anchored to the membrane by *bis*-phosphorylated lipid A (Figure 5) providing a negatively charged target for positively charged CAMPs.

**Figure 6:**
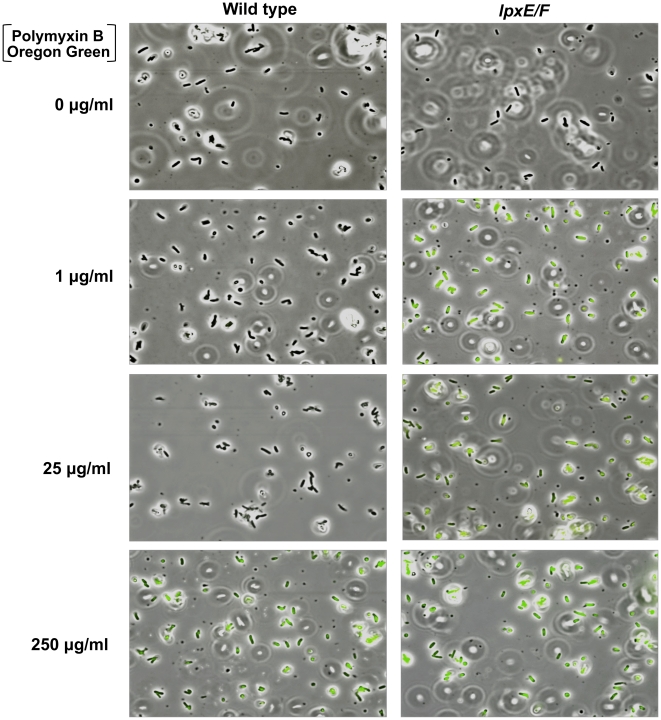
Fluorescent polymyxin B whole cell binding assay. *H. pylori* wild type J99 and the double *lpxE/F* mutant were incubated for 10 minutes in the presence of Oregon Green 514 polymyxin B (PMB-OG) at the indicated concentrations. Bacteria were visualized by phase contrast and fluorescence microscopy (shown as overlays, 1000X). Unlike wild type, the double *lpxE/F* mutant fluoresced when incubated with 1 or 25 µg/ml PMB-OG, suggesting increased ability to bind CAMPs. Both strains fluoresced when incubated with 250 µg/ml, although wild type fluorescence was lower in intensity. See Figure S3 in Text S1 for quantitative measure of this data.

To visually assess this, we devised a PMB binding assay by use of a biologically active fluorescent Oregon Green 514 derivative of polymyxin B (PMB-OG). Briefly, *H. pylori* wild type and double *lpxE/F* mutant in strain J99 were incubated for 10 minutes in the presence of 0, 1, 25, or 250 µg/ml PMB-OG and visualized by phase contrast and fluorescence microscopy (Figure 6). Overlay images clearly illustrate that unlike wild type, the double *lpxE/F* mutant fluoresced when incubated with 1 µg/ml PMB-OG, indicating increased surface-bound or entry of PMB-OG. Even at 25 µg/ml of peptide, essentially no fluorescence was observed for wild type bacteria. Both strains fluoresced when incubated with 250 µg/ml PMB-OG, although wild type was lower in intensity. These images visually demonstrate the protective effect lipid A dephosphorylation by LpxE and LpxF has against CAMP attack of the bacterial surface of *H. pylori*.

To quantify the binding and/or entry of PMB-OG to *H. pylori* strains, stained cells were re-suspended in PBS and fluorescent intensity measured (Figure S3 in Text S1). The double *lpxE/F* mutant showed significantly increased fluorescence when compared to wild type confirming our visual interpretation of the fluorescence microscopy. Furthermore, quantitative fluorescent analysis of PMB-OG binding to the *lpxE*, *lpxF*, *lpxR* and complemented mutants corresponded to the loss of CAMP resistance seen in our MIC experiments (Table 2) solidifying these findings.

### Dephosphorylation of Lipid A by LpxE and LpxF Results in Attenuated TLR4-MD2 Activation and Has No Effect on TLR2 Activation

*H. pylori* LPS is characterized by strikingly low endotoxicity which may contribute to the long-term carriage state of the organism [18]. LPS from *F. tularensis* and *P. gingivalis* also exhibit very low endotoxicity and both organisms harbor lipid A phosphatases [31], [32]. LPS purified from LpxF deficient *F. tularensis* mutants which produce a penta-acylated form of lipid A failed to show increases in hTLR4-MD2 activation, indicating no role in attenuation [31]. However, given that hTLR4-MD2 is unable to recognize LPS anchored to the membrane by a penta-acylated form of lipid A, this result is not surprising [33]. Alternatively, LPS purified from LpxE and/or LpxF deficient *P. gingivalis*, triggered an increased hTLR4-MD2 response, indicating lipid A dephosphorylation plays a role in its low endotoxicity [32]. The ability of LPS from a number of bacteria to elicit an immune response has been examined. However, much of the literature describes experiments using LPS isolated from bacteria (e.g. *P. gingivalis*) producing a mixture of lipid A species, complicating the findings [34]. Further confusing the literature are reports that heavily modified forms of lipid A, like that found in *H. pylori, P. gingivalis*, and *Leptospira interrogans* elicit an immune response through hTLR2 [14], [35], [36], [37], [38]. Fortuitously, *H. pylori* and the mutants generated herein produce an abundant single species of lipid A (Figures 4 & 5), giving us the ability to investigate the contribution of single lipid A modification in hTLR4-MD2 and hTLR2 activation.

**Figure 7:**
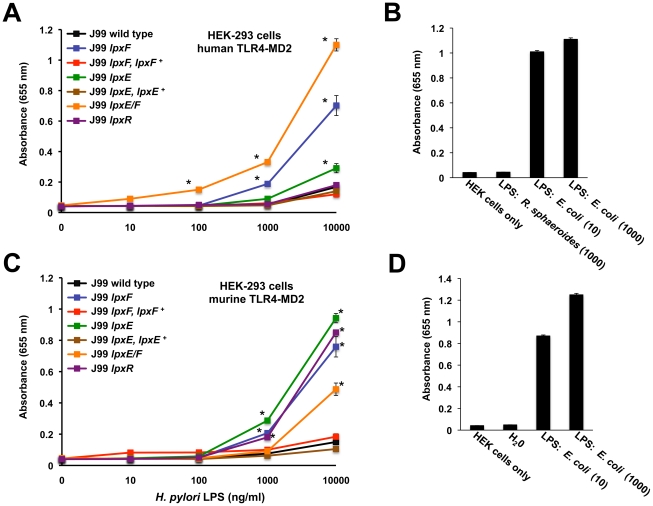
Activation of human or murine TLR4-MD2 by *H. pylori* LPS. Activation of TLR4 was monitored using HEK-293 cells stably transfected with either human TLR4-MD2-CD14 or murine TLR4-MD2-CD14. TLR activation was monitored colorimetrically using a secreted alkaline phosphatase (SEAP) reporter gene placed under the control of an NF-κB inducible promoter. Highly purified LPS from the indicated strains of *H. pylori* was added to each well in triplicate at 0, 10, 100, 1000 and 10000 ng/ml. Values are the mean of results from triplicate wells ± standard deviation. When compared to wild type, LPS (10000 ng/ml) from the *lpxE, lpxF,* and *lpxE/F* mutants showed an ∼2, 6 and 10-fold significant increase in hTLR4-MD2 activation, respectively. hTLR4-MD2 acitvation by LPS prepared from the LpxR mutant and complemented strains were identical to that of wild type (p>0.05). Where appropriate, an asterisk was used to indicate significance (p ≤0.001) (A). For experiments with hTLR4-MD2, LPS from *R. sphaeroides* (1000 ng/ml) and *E. coli* (10 and 1000 ng/ml) was used as negative and positive controls for activation of hTLR4-MD2, respectively (B). When compared to wild type, LPS (10000 ng/ml) from the *lpxE, lpxF, lpxR* and *lpxE/F* mutants showed an ∼10, 8, 9 and 5 fold significant increase in mTLR4-MD2 activation, respectively. mTLR4-MD2 acitvation by LPS prepared from complemented strains were identical to that of wild type (p>0.05). Where appropriate, an asterisk was used to indicate significance (P ≤0.01) (C). For experiments with mTLR4-MD2, endotoxin free water and LPS purified from *E. coli* (10 and 1000 ng/ml) was used as negative and positive controls for activation of mTLR4-MD2, respectively (D). LPS from *R. sphaeroides* is a known mTLR4-MD2 agonist and was not used as a negative control. All results agreed between experimental and biological replicates. Cells were stimulated for 24 hours with the indicated ligands.

The Toll-activation profiles of intact LPS were examined using samples purified using by the Hirschfield method [39] that allows for removal of potential contaminating lipoproteins. Activation of TLRs was monitored using HEK-Blue 293 cells stably transfected with TLR machinery and a secreted alkaline phosphatase (SEAP) reporter gene placed under the control of an NF-κB inducible promoter allowing for easy detection of TLR activation using a colorimetric assay. Changes in hTLR4-MD2 activation in the *H. pylori* strains are clearly illustrated in Figure 7A. LPS from the *H. pylori* single *lpxE*, *lpxF* and double *lpxE/F* mutant show 2, 6 and 10 fold significant (P ≤0.001) increases in activation of hTLR4-MD2 at 10000 ng/ml when compared to wild type, indicating that LpxF imparts greater influence on attenuated hTLR4-MD2 when compared to LpxE. This agrees with the published crystal structure of the hTLR4-MD2-LPS complex showing that the 4′ phosphate group is involved for ligand recognition [40]. Similar results were seen using 1000 ng/ml of LPS (Figure 7A). LPS isolated from the *lpxR* deficient mutant activated hTLR4-MD2 similar to wild type LPS indicating that lipid A dephosphorylation plays a larger role in lowering endotoxicity compared to deacylation of *Helicobacter* lipid A (Figure 7A). Although these findings are of interest, it is important to note that in comparison to *E. coli* LPS, higher concentrations of *Helicobacter* LPS are required for activation of TLR4. For each experiment LPS from *R. sphaeroides* (1000 ng/ml), a known TLR4 antagonist [41], and *E. coli* (10 and 1000 ng/ml) were used as a negative and positive controls for activation of hTLR4-MD2, respectively (Figure 7B).

**Figure 8:**
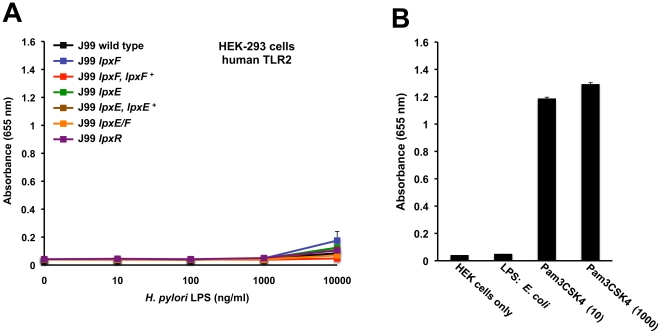
Activation of hTLR2 by *H. pylori* LPS. Activation of hTLR2 was monitored using HEK-293 cells stably transfected with hTLR2. TLR activation was monitored colorimetrically using a secreted alkaline phosphatase (SEAP) reporter gene placed under the control of an NF-κB inducible promoter. Cells were stimulated for 24 hours with the indicated ligands. Highly purified LPS from the indicated strains of *H. pylori* was added to each well in triplicate at 0, 10, 100, 1000 and 10,000 ng/ml. Values are the means of results from triplicate wells ± standard deviation. No significant activation of hTLR2 was seen in LPS prepared from the *lpxE, lpxF,* and *lpxE/F* mutants when compared to wild type *H. pylori* J99 (P>0.05) (A). The results agreed between experimental and biological replicates. For each experiment LPS from *E. coli* (1000 ng/ml) and Pam3CSK4 (10 and 1000 ng/ml), a synthetic lipopeptide, were used as negative and positive controls for activation of hTLR2 (B), respectively. Identical results were seen for activation of mTLR2 (data not shown).

LPS from the *H. pylori* single *lpxE*, *lpxF* and double *lpxE/F* mutants showed no significant activation of hTLR2 (p>0.05), even at LPS concentrations as high as 10,000 ng/ml when compared to wild type LPS (Figure 8A). LPS from *E. coli* (1000 ng/ml) and Pam3CSK4 (10 and 1000 ng/ml), a synthetic lipopeptide, were used as a negative and positive control for activation of hTLR2, respectively (Figure 8B). *H. pylori* LPS has been reported to act as both a hTLR4-MD2 and hTLR2 agonist [42]; however, the ability of *H. pylori* LPS to activate hTLR2 is more controversial. Our data suggest that *H. pylori* LPS does not activate hTLR2. Some of these discrepancies may arise because of contaminating lipoproteins in LPS preparations. Recent literature documenting activation of hTLR2 by *H. pylori* LPS used concentrations as high as 10,000 ng/ml [42], increasing the possibility that activation was not the result of LPS but rather contamination. All TLR activation studies were performed using LPS purified from strains in background J99.

Previous research has demonstrated that hTLR4-MD2 and murine TLR4-MD2 (mTLR4-MD2) display differential recognition of LPS [33]. In anticipation of a future colonization study using a murine host, we felt it necessary to address this by examining the ability of *H. pylori* LPS to activate mTLR4-MD2. Activation of TLRs was monitored using HEK-Blue 293 cells stably transfected with murine TLR machinery using the same reporter system described above. Changes in mTLR4-MD2 activation in the *H. pylori* strains are clearly illustrated in Figure 7C. LPS from the *H. pylori* single *lpxE*, *lpxF*, *lpxR* and double *lpxE/F* mutant show 10, 8, 9 and 5 fold significant (p ≤0.001) increases in activation of mTLR4-MD2 at 10000 ng/ml when compared to wild type (Figure 7B), suggesting that LpxE, LpxF and LpxR all play similar roles in mTLR4-MD2 ligand recognition and confirming species-specific differential recognition of LPS. Differential recognition by mTLR4-MD2 of LPS purified from an *lpxR* deficient background is not surprising considering the promiscuity of this receptor for lipid A isoforms (i.e. tetra- and penta-acylated lipid A) [33]. For each experiment endotoxin free water and *E. coli* (10 and 1000 ng/ml) were used as negative and positive controls for activation of mTLR4-MD2, respectively (Figure 7D). LPS from the *H. pylori* single *lpxE*, *lpxF, lpxR* and double *lpxE/F* mutants showed no significant activation of mTLR2 (p>0.05), even at LPS concentrations as high as 10,000 ng/ml when compared to wild type LPS (data not shown).

### Dephosphorylation of Lipid A by LpxF Is Required for Host Colonization in *H. pylori*

To date, nothing is known regarding participation of *H. pylori* LPS modifications in host colonization. Presumably, dephosphorylation by LpxE and LpxF provides an advantage through resistance to CAMPs at the gastric mucosal surface and attenuated hTLR-MD2 activation. *Francisella* mutants lacking *lpxF* were greatly attenuated in animal studies and considered avirulent [31]. However, *F. tularemia* does not establish a long-term colonization of its human host but rather, results in quick progression to severe illness and/or death [43]. Considering our current findings, we felt it was important to proceed with an *in vivo* animal study to emphasize the role played by LpxE and LpxF in host colonization.

For these experiments, all mutations were transferred into the mouse-adapted strains B128 and X47 [24]. Two bacterial strains were used to rule out the influence of variation between strains and for confirmation of findings. Recent publications in *Campylobacter jejuni* and *H. pylori* have demonstrated that lipid A modification enzymes can modulate motility and O-antigen expression, respectively [19], [44]. Both of these have been shown to be important for host colonization. Given these findings, we felt it important to examine both phenotypes prior to colonization studies. The LPS profiles (O-antigen polysaccharide and core oligosaccharide composition) of all mutants were indistinguishable from that of wild type during SDS-PAGE (Figure S4 in Text S1) and all strains showed normal motility in a soft agar assay (Figure S5 in Text S1), suggesting LpxE and LpxF do not influence these phenotypes. Mutants in *H. pylori* background J99 were used to illustrate these findings. However, identical results were found in both mouse-adapted strains, B128 and X47 (data not shown).

Two mice populations, C57BL/6J and C57BL/6J *tlr*4^-^/^-^, were colonized. The TLR4 deficient mice were used in conjunction with wild type mice to help determine which plays a larger role in host colonization: CAMP resistance or attenuated hTLR4-MD2 activation. Mice were infected orogastrically with 2×10^8^ bacteria per mouse. Colonization rates were determined by enumeration of colony forming units per gram (CFU/g) of stomach. Two independent colonization experiments were performed. The first was a 15-day time point using both B128 and X47 strains in C57BL/6J. The second was a time course colonization experiment at days 3, 15, and 45 using only strain B128 in both C57BL/6J and C57BL/6J *tlr*4^-^/^-^ mice.

**Figure 9:**
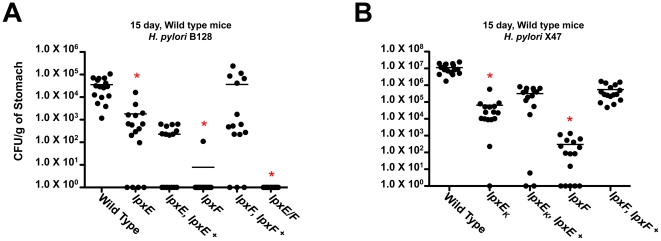
*In vivo* colonization of the *lpxE, lpxF* and *lpxE/F* mutants in strain B128 and X47 at day 15 using C57BL/6J mice. C57BL/6J mice were infected orogastrically with the indicated strains at 2×10^8^ bacteria per mouse. Colonization rates were determined after 15 days by enumeration of colony forming units per gram (CFU/g) of stomach. Circles represent individual mice while mean colonization levels are denoted by horizontal bars. Circles located on the x-axis represent mice with no colonization. The single *lpxE*, *lpxF* and double *lpxE/F* mutants in background B128 (A) and single *lpxE* and *lpxF* mutants in background X47 (B), showed a statistically significant colonization defect when compared to wild type as indicated by a red asterisk (p<0.0001). In B128, complementation of LpxE deficient strains failed to restore the colonization defect. However, complementation of the LpxF deficient strain successfully restored colonization to wild type levels (p>0.05). In X47, complementation of LpxE and LpxF deficient strains resulted in partial recovery of the colonization defect but still significantly different from wild type (p<0.05). Data from two independent cohorts of mice at day 15 were combined to increase significance and robustness of our analysis.

Results from the first experiments revealed that in wild type C57BL/6J mice the single *lpxE*, *lpxF* and double *lpxE/F* mutants, in background B128 and X47, showed a statistically significant colonization defect (p<0.0001) (Figure 9A and 9B). In B128, complementation of *lpxE* deficient strains failed to restore the colonization defect. However, complementation of the *lpxF* deficient strain successfully restored colonization to wild type levels (p>0.05). In X47, complementation of *lpxE* and *lpxF* deficient strains resulted in partial recovery of the colonization defect but still significantly different from wild type (p<0.05). Data from both cohorts of mice at day 15 were combined to increase significance and robustness of our analysis. The double *lpxE/F* mutant in strain X47 was not included because the addition of a chloramphenicol cassette shows a strain specific decrease in the fitness of the bacteria in colonization experiments (unpublished observation). Overall, *lpxF* deficient strains in both B128 and X47 background showed the largest defect in colonization, suggesting LpxF activity is essential for host colonization.

**Figure 10:**
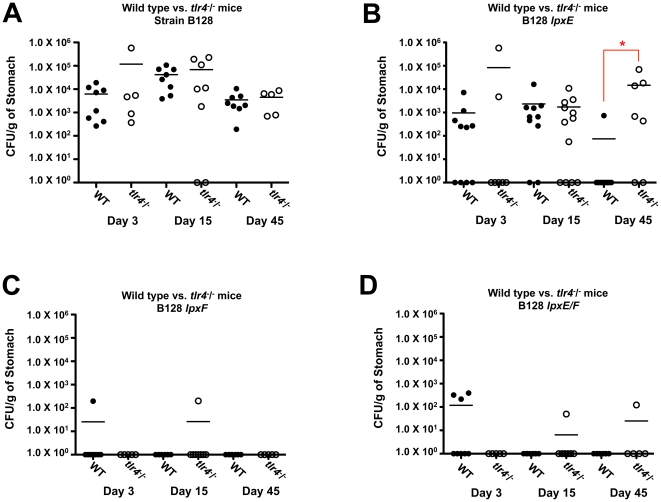
A comparision of *in vivo* colonization of the *lpxE, lpxF* and *lpxE/F* mutants in strain B128 using both C57BL/6J and C57BL/6J *tlr*4^-^/^-^ mice. C57BL/6J and C57BL/6J *tlr*4^-^/^-^ mice were infected orogastrically with the indicated strains at 2×10^8^ bacteria per mouse. Colonization rates were determined after 3,15 and 45 days by enumeration of colony forming units per gram (CFU/g) of stomach. Closed circles represent individual C57BL/6J mice and open circles represent individual C57BL/6J *tlr*4^-^/^-^ mice. Mean colonization levels are denoted by horizontal bars. Circles located on the x-axis represent mice with no colonization. Wild type strain B128 colonized C57BL/6J and C57BL/6J *tlr*4^-^/^-^ mice equally well (p>0.05) throughout the time course (A). Single LpxF (C) and double LpxE/F (D) deficient strains were mostly unable to colonize C57BL/6J or C57BL/6J *tlr*4^-^/^-^ mice mice, demonstrating colonization defects by day 3 and no significant differences were seen when comparing C57BL/6J and C57BL/6J *tlr*4^-^/^-^ mice (p>0.05). However, the LpxE (B) deficient strain colonized well until day 45 and a significant difference was seen when comparing C57BL/6J and C57BL/6J *tlr*4^-^/^-^ mice (p<0.01) as indicated by an asterisk.

A second colonization experiment was performed using B128 strains in C57BL/6J and C57BL/6J *tlr*4^-^/^-^ mice. A 3, 15 and 45 day time course was included in this study to determine if *H. pylori* strains were cleared soon after infection or if it was a gradual loss of colonization. Wild type strain B128 colonized C57BL/6J and C57BL/6J *tlr*4^-^/^-^ mice equally well (p>0.05) throughout the time course (Figure 10A). Single LpxF and double LpxE/F deficient strains were mostly unable to colonize C57BL/6J or C57BL/6J *tlr*4^-^/^-^ mice mice, demonstrating a colonization defect by day 3 (Figure 10C and 10D) and no significant differences were seen when comparing C57BL/6J and C57BL/6J *tlr*4^-^/^-^ mice (p>0.05). However, the LpxE deficient strain colonized well until day 45 (Figure 10B) and a significant difference was seen when comparing C57BL/6J and C57BL/6J *tlr*4^-^/^-^ mice (p<0.01). This suggests that resistance to CAMPs, resulting from the dephosphorylation of lipid A by LpxF, is important for survival within a host. The role LpxF dependent attenuated TLR4-MD2 activation plays in the colonization of a host cannot be determined because they are so quickly cleared from the mouse stomach, presumably due to CAMP sensitivity. However, LpxE deficient strains retain partial resistance to CAMPs (Table 2), giving us the ability to compare mTLR4-MD2 dependent colonization. Interestingly, a colonization defect seen at day 45 using wild type mice disappears in *tlr*4^-^/^-^ mice. Given that the LPS from *lpxE* mutants shows a 10-fold increase in activation of mTLR4-MD2 (Figure 7C), one can speculate that dephosporylation of lipid A by LpxE results in attenuated TLR4-MD2 activation required for long term colonization of a host. Taken together, these findings indicate that dephosphorylation of lipid A by LpxE and LpxF in *H. pylori* plays a role in effective colonization and survival within a host.

## Discussion

The primary surface component of nearly all gram-negative bacteria is the glycolipid LPS. LPS is localized to the outer leaflet of the outer membrane, providing an additional layer of protection from the external environment and interfacing with the surrounding environment. Modification of the bioactive Kdo-lipid A domain of LPS is a common theme among gram-negative pathogens, most likely providing an advantage to the invading pathogen [6]. *H. pylori* is a great example, producing a highly modified form of lipid A that aids in camouflaging the bacterium from the innate immune response of its host allowing it to set up a life long chronic infection of the gastric mucosa.

The identification and characterization of LpxF in this work completes the five-step enzymatic pathway by which *H. pylori* lipid A is modified (Figure 2), resulting in an outer surface that is highly resistant to CAMPs and LPS that shows attenuated TLR4-MD2 activation [19]. Given that deletion of some enzymes in this five-step pathway (e.g. LpxE, KdoH1/2, and LpxF), result in the complete or partial loss of “downstream” modifications, the order for modification of *H. pylori* lipid A can now be determined (Figure 2). A recent publication demonstrated that efficient ligation of O-antigen to the core-lipid A molecule is modulated by KdoH1/2 activity in *H. pylori*, suggesting that surface LPS glycosylation is also an ordered process linked to lipid A modification [19]. Thus, it appears that the enzymatic machinery responsible for the maturation of LPS in *H. pylori* evolved a highly ordered pathway, ensuring a single and very specific surface-exposed LPS that is essentially invisible to its host innate immune system. Moreover, the only known variable region of the *H. pylori* LPS molecule is the O-antigen, most likely allowing for adaptation to antigenic variation in host cell surfaces [4]. One could speculate that lack of multiple hosts for *H. pylori* was the key evolutionary pressure leading to an ordered pathway; hence, any genetic drift resulting in changes to the lipid A structure were quickly selected against by the host innate immune system.

CAMP resistance is important for any pathogen, especially those that establish long-term colonization of a host and *H. pylori* is an excellent example. Our research identifies the dephosphorylation of lipid A in *H. pylori* by LpxE and LpxF as the most important determinant of CAMP resistance identified to date in this organism. Furthermore, our data suggest that although both LpxE and LpxF act synergistically to provide resistance to CAMPs, LpxF imparts the most influence. The removal of a Kdo core sugar by KdoH1/2 was shown to influence CAMP resistance in previous studies, with KdoH1/2 deficient strains having a polymyxin B MIC of between 0.5 and 1.0 µg/ml, similar to what is seen in LpxF deficient strains [19]. This can be attributed to the inefficient removal of the 4′-phosphate group by LpxF in KdoH1/2 deficient strains of *H. pylori*.

It is well documented that removal or “masking” of phosphate groups is the primary feature involved in CAMP resistance in several organisms [6]. However, the exact mechanism for resistance is more speculative and thought to occur by reducing the binding efficiency of these peptides. By use of a biologically active fluorescent PMB, our data demonstrates that membranes composed of fully phosphorylated lipid A show an increased ability to bind CAMPs. Moreover, the use of biologically relevant CAMPs found at multiple locations within a human host provides evidence that resistance to CAMPs is not only important for survival at the site of colonization, but also during transmission of *H. pylori* through the oral cavity.

LPS serves as one of the most powerful activators of the innate immune system. Presumably, *H. pylori* must avoid activation of hTLR4-MD2 to set up long-term colonization of the gastric mucosa. Producing an LPS characterized by strikingly low endotoxicity likely contributes to this immune deception. But which lipid A modifications in *H. pylori* have the greatest impact on attenuated hTLR4-MD2 activation? Our data suggest that dephosphorylation of lipid A by LpxE and LpxF contributes to the low endotoxicity of *H. pylori* LPS, but is not the only factor. It should be noted that LPS prepared from the double LpxE/F mutant, synthesizing lipid A that is *bis*-phosphorylated and hexa-acylated, still shows attenuated hTLR4-MD2 activity when compared to *bis*-phosphorylated hexa-acylated lipid A of LPS prepared from *E. coli* K-12 stains (Figure 7), requiring high concentrations of LPS (100 ng/ml) for *in vitro* activation of hTLR4-MD2. It is likely these differences arise from the increased length of acyl chains (16 and 18 carbons) found on *H. pylori* lipid A. Additionally, our data suggest that *H. pylori* LPS does not activate hTLR2 even at high concentrations of LPS, helping to clarify some conflicting reports in the literature [42].

Previous to this work, nothing was known regarding participation of *H. pylori* lipid A in host colonization. This work clearly demonstrates that dephosphoryation of *H. pylori* lipid A by LpxE and LpxF is essential for efficient host colonization, with LpxF activity being the largest determinant of bacterial survival within a host. LpxF deficient strains are unable to colonize host and are quickly cleared within 3 days of inoculation (Figure 10A and 10B), presumably due to loss of CAMP resistance (Table 2). However, LpxE deficient strains retain partial resistance to CAMPs (Table 2) and show longer-term survival within a murine model but are cleared in a mTLR4-MD2 dependent manner by day 45 (Figure 10B). Given that LPS isolated from LpxE deficient strains show a 10-fold increase in mTLR4-MD2 activation (Figure 7B), mTLR4-MD2 dependent clearance from the murine stomach seems likely. Furthermore, considering the 10-fold increase in hTLR4-MD2 seen with LPS purified from LpxF deficient strains, an even greater role in immune evasion within the human stomach is suggested and highlights the importance of lipid A modification in the pathogenesis of *H. pylori*.

To date, all LpxF enzymes identified only catalyze the removal of the 4′-phosphate group from penta-acylated forms of lipid A [31], [45]. *H. pylori* LpxF differs in that it can dephosphorylate hexa-acylated forms of lipid A (Figure 3). The use of *H. pylori* LpxF for the synthesis of 4′-monophosphoryl lipid A variants could prove useful in the development of vaccine adjuvants. Lipid A derivatives that lack the 1-phosphate group can be prepared by chemical synthesis, acid hydrolysis of LPS, or LpxE phosphatase treatment and are referred to as 1-monophosphoryl lipid A [46], [47], [48], [49]. More recently, some 4′-monophosphoryl lipid A variants have been approved as adjuvants for use in human vaccines. These lipid A variants retain full adjuvant activity while demonstrating low endotoxicity [50], [51].

In this research, we identify the enzyme responsible for lipid A 4′-phosphatase activity as Jhp1487 (LpxF) in *H. pylori*. We also demonstrate that dephosphorylation of lipid A by LpxE and LpxF in *H. pylori* provides resistance to CAMPs and results in attenuated hTLR4-MD2 activation, with LpxF having the largest impact in both cases. Removal of phosphate groups from the lipid A backbone is essential for colonization of *H. pylori* in a mammalian host. This research highlights the importance of membrane lipid composition in the pathogenesis of gram negative bacteria and provides clear evidence that lipid A is an important virulence factor.

## Materials and Methods

### Ethics Statement

This study was carried out in strict accordance with the European Union Directive 2010/63/EU (and its revision 86/609/EEC) on the protection of animals used for scientific purposes. Our laboratory has the administrative authorization for animal experimentation (Permit Number 75–1250) and the protocol was approved by the Institut Pasteur Review Board that is part of in the Regional Committee of Ethics of Animal Experiments of Paris region (Permit Number: 99–174).

### Bacterial Strains and Growth Conditions

The bacterial strains and plasmids used in the study are summarized in Table 3. Cultures of *H. pylori* were started from methyl-cellulose stocks routinely stored at −80°C, plated onto 5% sheep blood agar medium (BAP) and incubated at 37°C for 36 to 60 h in a microaerobic atmosphere (5% O_2_, 10% CO_2_, 85% N_2_). The methyl-cellulose storage media is composed of cellulose-methyl ether 4000 CPS grade (1.0% w/v) and MgSO4 (0.1 M) and the BAP is composed of Tryptic soy agar (acumedia Cat. 7100A) and defibrinated sheep blood (Remel Cat. R54020). Liquid cultures of *H. pylori* were started from overnight culture on BAP and inoculated into brucella broth supplemented with 7% fetal bovine serum (Hyclone) and vancomycin (10 µg/ml). Liquid cultures of *H. pylori* were grown to an *A*_600_ of ∼1.0 at 37°C under microaerobic conditions for 24 to 36 h with shaking. *E. coli* was routinely grown at 37°C in LB broth with appropriate antibiotics using standard conditions.

**Table 3:** Bacterial strains and plasmids used In this study.

Strain	Genotype or description^a^	Reference
*E. coli*		
XL-1 Blue	General Cloning Strain, *recA1 endA1 gyrA96thi-1 hsdR17 supE44 relA1 lac* [F' *proAB lacI^q^*ZM15::Tn*10*], Tet^R^	Stratagene
***H. pylori***		
J99	Wild Type	ATCC700824
B128	Wild Type	[23]
X47	Wild Type	[24]
J99 *lpxF*	J99 with kanamycin resistance cassette in *jhp1487,* Kan^R^	This study
J99 *lpxF, lpxF* ^+^	J99 *lpxF* with *jhp1487* in *rdxA*, Kan^R^, Met^R^	This study
J99 *lpxE*	J99 with chloramphenicol resistance cassette in *jhp0019,* Cam^R^	[16]
J99 *lpxE, lpxE* ^+^	J99 *lpxE* with *jhp0019* in *rdxA*, Cam^R^, Met^R^	This study
J99 *lpxE/F*	J99 *lpxF* with chloramphenicol resistance cassette in *jhp0019*, Kan^R^, Cam^R^	This study
J99 *lpxR*	J99 with chloramphenicol resistance cassette in *jhp0634,* Cam^R^	[21]
B128 *lpxF*	B128 with kanamycin resistance cassette in *jhp1487,* Kan^R^	This study
B128 *lpxF, lpxF* ^+^	B128 *lpxF* with *jhp1487* in *rdxA*, Kan^R^, Met^R^	This study
B128 *lpxE*	B128 with chloramphenicol resistance cassette in *jhp0019,* Cam^R^	This study
B128 *lpxE, lpxE* ^+^	B128 *lpxE* with *jhp0019* in *rdxA*, Cam^R^, Met^R^	This study
B128 *lpxE/F*	B128 *lpxF* with chloramphenicol resistance cassette in *jhp0019*, Kan^R^, Cam^R^	This study
X47 *lpxF*	X47 with kanamycin resistance cassette in *jhp1487,* Kan^R^	This study
X47 *lpxF, lpxF* ^+^	X47 *lpxF* with *jhp1487* in *rdxA*, Kan^R^, Met^R^	This study
X47*lpxE*	X47 with chloramphenicol resistance cassette in *jhp0019,* Cam^R^	This study
X47 *lpxE, lpxE* ^+^	X47 *lpxE* with *jhp0019* in *rdxA*, Cam^R^, Met^R^	This study
X47*lpxE_K_*	X47 with kanamycin resistance cassette in *jhp0019,* Kan^R^	This study
X47 *lpxE_K_, lpxE* ^+^	X47 *lpxE* with *jhp0019* in *rdxA*, Kan^R^, Met^R^	This study
X47 *lpxE/F*	X47 *lpxF* with chloramphenicol resistance cassette in *jhp0019*, Kan^R^, Cam^R^	This study
**Plasmids**		
pBluescript II SK(+)	High-copy Cloning Vector, Amp^R^	Stratagene
pET21a	High-copy Cloning vecotor containing a T7 promoter, Amp^R^	Novagen
piLLhp1580:kan	piLL570 with *hp1580* interrupted with kanamycin resistance cassette, Spec^R^	This study
pET634comp	pET21a with *jhp0634* inserted into *hp0954*, Amp^R^	[21]
pETjhp1487comp	pEThp0954 with *jhp1487* inserted into *hp0954*, Amp^R^	This study
pETjhp0019comp	pEThp0954 with *jhp0019* inserted into *hp0954*, Amp^R^	This study
pUC18-Km2	pUC18 containing a kanamycin resistance cassette from *H. pylori*, Amp^R^	[53]
piLLhp1580	piLL570 with DNA fragment containing *hp1580*, Spec^R^	[52]

a Antibiotics were used at the following concentrations:

For *E. coli* cultures, 100 µg/ml ampicillin (Amp^R^), 100 µg/ml spectinomycin (Spec^R^). For *H. pylori* cultures, 8 µg/ml chloramphenicol (Cam^R^), 8 µg/ml kanamycin (Kan^R^), and 10 µg/ml metronidazole (Met^R^).

### Recombinant DNA Techniques

Plasmids were isolated using a QIAprep Spin Miniprep Kit (Qiagen). Custom primers (Table S1 in Text S1) were obtained from Integrated DNA Technologies. PCR reagents were purchased from Stratagene. PCR clean up was performed using a Qiaquick PCR Purification Kit (Qiagen). DNA fragments were isolated from agarose gels using a Qiaquick Gel Extraction Kit (Qiagen). Restriction endonucleases, T4 DNA ligase, and antarctic phosphatase were purchased from New England Biolabs. All modifying enzymes were used according to the manufacturer's instructions.

### Construction of *H. pylori* Mutants

To construct the lipid A 4′-phosphatase mutant, plasmid pILL570 containing *hp1580* (*lpxF*), from *H. pylori* 26695 was obtained from a previously published library [52]. This plasmid was reverse amplified using primers 5 and 6 (Table S1 in Text S1), incorporating BamH1 and Kpn1 restriction sites and leaving 300 bps at the 5′ and 3′ end of the gene. A non-polar kanamycin cassette was digested out of plasmid pUC18-Km2 [53] using BamH1 and Kpn1 and ligated into the reverse PCR product in the same orientation as *hp1580* using standard cloning techniques. The generated plasmid, pILLhp1580:kan, was used to create 4′-phosphatase defective mutants in *H. pylori* J99, B128 and X47 by natural transformation [54]. Resistant colonies were selected on blood agar plates containing 8 µg/ml of kanamycin, patched onto kanamycin-containing plates, and verified by PCR of genomic DNA. Gene *hp1580* corresponds to *jhp1487* in *H. pylori* J99. Mutation of *jhp0019* (*lpxE*) and *jhp0634* (*lpxR*) in *H. pylori* B128 and X47 was achieved by introduction of a chloramphenicol or kanamycin cassette into the coding sequence of each gene resulting in the interruption and removal of a large portion of its sequence, using previously described suicide vectors [17], [22]. The *lpxE*/*F* double mutant was created by introduction of the *lpxE* mutation into a *lpxF* defective mutant background using natural transformation as described above.

### Chromosomal Complementation of *H. pylori lpxE* and *lpxF* Defective Mutants

The interruption of *hp0954* (*rdxA*) renders *H. pylori* resistant to metronidazole making it an ideal candidate for chromosomal complementation [55]. Using methods previously described [22], *lpxE* and *lpxF* defective mutants were complemented by insertion and consequent disruption of *rdxA.* Briefly, the *lpxE* and *lpxF* genes plus 500 bps upstream were amplified by PCR (primers 3,4 and primers 1,2, respectively), Table S1 in Text S1, from *H. pylori* J99 genomic DNA using *Pfu* Turbo (Stratagene) according to the manufacturer's instructions. The *lpxE* and *lpxF* amplicons were digested with BamH1 and EcoR1, gel purified, and subsequently ligated into the previously described *H. pylori rdxA* complementation plasmid pET634comp [22]. Previous to ligation, plasmid pET634comp was digested with BamH1 and EcoR1, gel purified, and treated with antarctic phosphatase. The resulting plasmids pETjhp1487comp and pETjhp0019comp were transformed into *H. pylori* J99 *lpxE* and *lpxF* deficient mutants by natural transformation [54]. The same method was used for complementation in X47 and B128 backgrounds. Resistant colonies were selected on blood agar plates containing 12 µg/ml metronidazole, patched onto metronidazole-containing plates, and verified by PCR of genomic DNA.

### Isolation and Analysis of Lipid A Species from ^32^P_i_-Labelled Cells

*H. pylori* (25 ml) and *E. coli* (5 ml) cultures were grown in the presence of 5.0 µCi/ml ^32^P_i_ (PerkinElmer) to an A_600_ of ∼1.0 using standard liquid growing conditions described above and the cells harvested by centrifugation. ^32^P-labelled lipid A and phospholipids were isolated using published protocols [20] and spotted onto a Silica Gel 60 TLC plate (EMD) at 2500 cpm/lane. Lipid species were separated using the solvent chloroform, pyridine, 88% formic acid and water (50∶50∶16∶5, v/v). The TLC plates were exposed overnight to a phosphorimager screen and visualized using a Bio-Rad Molecular Imager phosphorimager equipped with Quantity One software.

### Isolation of Lipid A for Mass Spectrometry Analysis

*H. pylori* cultures (25 ml) were grown to an A_600_ of ∼1.0 using standard liquid growing conditions described above and the cells harvested by centrifugation. Lipid A was purified from bacteria as described previously [20] and stored frozen at −20 °C. The lipid A species were analyzed using a MALDI-TOF (ABI Voyager-DE PRO) mass spectrometer equipped with a N_2_ laser (337 nm) using a 20 Hz firing rate. The spectra were acquired in negative ion reflectron mode and each spectrum represented the average of a minimum of 4000 shots. The matrix used was a saturated solution of 6-aza-2-thiothymine in 50% acetonitrile and 10% tribasic ammonium citrate (9∶1, v/v). The samples were dissolved in chloroform-methanol (4∶1, v/v) and deposited on the sample plate, followed by an equal portion of matrix solution (0.3 µl).

### Preparation of Cell-free Extracts, Double-spun Cytosol, and Washed Membrane

*H. pylori* cultures (200 ml) were harvested at A_600_ of ∼1.0 and the cells harvested by centrifugation. Cell free extracts, membrane-free cytosol, and washed membranes were prepared as previously described [19] and were stored in aliquots at −20°C. Protein concentration was determined by the bicinchoninic acid method [56].

### Assay of the 4′-phosphatase Activity

The 4′-phosphatase activity of Jhp1487 (LpxF) was assayed under optimized conditions in a 10- µl reaction mixture containing 50 mM MES, pH 6.0, 0.2% Triton X-100, and Kdo_2_-[4′-^32^P]lipid A (at ∼3000–5000 cpm/nmol) as the substrate. Kdo_2_-[4′-^32^P]lipid A was prepared as previously described [21]. Washed membranes (1.0 mg/ml) were employed as the enzyme source. The dephosphorylation reaction was allowed to proceed at 30 °C for 3.0 h. Enzymatic reactions were terminated by spotting 4.5 µl of the mixtures on Silica Gel 60 TLC plates and the plate was dried under a cool air stream for 20 min. Reaction products were separated using the solvent chloroform, pyridine, 88% formic acid, water (30∶70∶16∶10, v/v). Reaction products were detected and analyzed using a Bio-Rad Molecular Imager PhosphorImager equipped with Quantity One Software.

### Fluorescent Polymyxin B Binding Assay

Liquid cultures of *H. pylori* were grown to an *A*_600_ of ∼0.8 to 1.0 at 37°C under microaerobic conditions for 24 to 36 h. The cells were washed with PBS (3X) followed by dilution to *A*_600_ of 0.05 in PBS. Polymyxin B Oregon Green 514 conjugate (Invitrogen) was added to 50 µl diluted cells at select concentrations and incubated at 37°C for 10 minutes. Following the incubation cells were washed with PBS (3X), resuspended in 50 µl of PBS, 15 µl placed onto poly-L-lysine coated slides (Electron Microscopy Sciences), and coverslips added. Bacteria were viewed at a magnification of 1000X with a Nikon Eclipse 80i microscope equipped with a 100x 1.4NA PLAN APO lens, a GFP band pass emission filter set with a 480±15 nm excitation range, a 535±20 nm emission range and a Photometrics Cool SNAP HQ^2^ camera. NIS-Elements AR 3.0 software was used to capture and create the merged images. To quantify PMB-OG binding, strains were cultured and stained as described above but in a volume of 200 µl. After washing, the cells were resuspended in PBS and placed into 96-well plates for analysis. Fluorescence (480 nm excitation and 535 nm emission) and *A*_600_ of each well was determined using a Synergy Mx multi-mode microplate reader (BioTek). Each experiment was repeated in triplicate and data reported as a ratio of Fluorescence intensity to *A*_600_. The results of three experiments were pooled and a one tailed Mann–Whitney test was used to determine statistical significance of observed differences (GraphPad Prism v5.0; GraphPad Software, CA).

### LPS Preparation

LPS from *H. pylori* was isolated from a 500 ml liquid culture grown under standard conditions to an *A*_600_ of ∼1.0 using the hot water-phenol method [57]. Isolated LPS was further purified to remove contaminating immuno-stimulatory proteins (e.g. lipoproteins) that could falsely alter TLR assays using the previously described Hirschfeld method [39]. Purified LPS was quantified using a Mettler Toledo XS105 Dual Range analytical balance (sensitivity≥0.1 ng) and resuspended in HyPure Cell Culture Grade Endotoxin Free Water (HyClone) to a concentration of 5 mg/ml.

### Determination of Minimum Inhibitory Concentrations (MIC)

MIC determination using Polymyxin B Etest strips (Biomérieux) was determined as previously described [19]. For MIC determination in liquid medium, the Hancock Laboratory Microtiter Broth Dilution Method was used and modified as needed [58]. *H. pylori* strains were grown overnight in brucella broth supplemented with 7% fetal bovine serum. The overnight cultures were inoculated into a 96-well microtiter plates at a starting *A*_600_ of 0.05, in a total volume of 100 µl standard *H. pylori* growing medium, supplemented with select concentration of CAMPs. The 96-well plates were incubated at 37°C in a microaerobic atmosphere (5% O_2_, 10% 1 CO_2_, and 85% N_2_) with constant shaking and *A*_600_ of each well determined using a Synergy-Mx monochromator based multi-mode microplate reader at 24 and 48 hours. Each experiment was repeated in triplicate. A positive growth control containing no CAMP and a negative control containing no bacteria was performed with every replicate. The MIC was taken as the lowest concentration of CAMP that reduced growth (*A*_600_) by more than 50% when compared to the positive growth control. The following CAMPs were purchased and used in the MIC experiment: polymyxin B (Sigma), human β defensin-2 (Phoenix Pharm), human cathelicidin LL-37 (Anaspec), histatin-5 P-113 (Sigma), human neutrophil peptide-2 (Bachem), and *H. pylori* peptide HP2-20 (Invitrogen). All peptides were stored according to manufacturers' instructions. Because of cost, MICs were only determined for mutants in the J99 background. Complemented strains were also excluded.

### TLR Signaling Assay

For TLR4 and TLR2, human epithelial kidney (HEK) 293 cells stably co-transfected with m or hTLR4, m or hMD2, and m or hCD14 (denoted as HEK-m/hTLR4) or m/hTLR2 and m/hCD14 (denoted as HEK-m/hTLR2) were purchased from InvivoGen (CAT. hkb-htlr4 and hkb-htlr2 or hkb-mtlr4 and hkb-mtlr2, respectively). All cell lines stably express secreted embryonic alkaline phosphatase (SEAP) under the control of a promoter inducible by NF-κB and activator protein 1 (AP-1). Thus, stimulation of m/hTLR4-MD2 or m/hTLR2 will result in an amount of extracellular SEAP in the supernatant that is proportional to the level of NF-κB induction. The cell lines were maintained in standard Dulbecco's modified Eagle's medium (DMEM) with 10% heat-inactivated fetal bovine serum (FBS) (Gibco) supplemented with 4.5 g/L glucose, 2 mM L-glutamine, 50 U/mL penicillin, 50 ug/ml streptomycin and 1X HEK-Blue selection (InvivoGen) in a 5% saturated CO_2_ atmosphere at 37°C.

The induction of TLR signaling in HEK-m/hTLR4 and HEK-m/hTLR2 cell lines was assessed by measuring SEAP activity using QUANTI-Blue^TM^ colorimetric assay (InvivoGen). The assay was performed according to manufacturer's protocols. Briefly, cells were seeded in a 96-well plate in triplicate (2.5×10^4^ cells/well for HEK-m/hTLR4 and 5×10^4^ cells/well for HEK-m/hTLR2) in the presence or absence of 10-fold dilutions of purified LPS. Controls included *Rhodobacter sphaeroides* LPS (TLR4 antagonist) (InvivoGen), *E. coli* W3110 LPS (TLR4 agonist) and the synthetic triacylated lipoprotein Pam3CSK4 (TLR2 agonist) (InvivoGen). After 20–24 h incubation, supernatants (20 ul) were transferred to a 96-well plate and incubated at 37°C with QUANTI-Blue (180 ul) for 1-2. SEAP activity was measured by reading optical density at 655 nm with a Synergy Mx multi-mode microplate reader (BioTek). The results of several (at least three) experiments were pooled and a one tailed Mann–Whitney test was used to determine statistical significance of observed differences (GraphPad Prism v5.0; GraphPad Software, CA).

### Mouse Colonization Assays

C57BL/6J female mice purchased from Charles River Laboratories and C57BL/6J *tlr4* -/- female mice from Pasteur Institute (kindly provided by Shizuo Akira) aged 4 to 5 weeks were infected orogastrically with feeding needles with either X47 or B128 strains (2×10^8^ bacteria per mouse). Colonization rates were determined after 3, 15 or 45 days by enumeration of colony forming units (CFU) per gram of stomach. Mice were euthanized with CO_2_ and the stomachs were ground and homogenized in peptone broth. The samples were then diluted and spread on blood agar plates supplemented with 200 µg/ml of bacitracin, and 10 µg/ml of nalidixic acid, to inhibit the growth of resident bacteria from the mouse forestomach. The CFUs were enumerated after 8 days of incubation under microaerobic conditions. The results of several (at least two) colonization experiments were pooled and a one tailed Mann–Whitney test was used to determine statistical significance of observed differences (GraphPad Prism v5.0; GraphPad Software, CA).

## Supporting Information

Text S1 *H. pylori* LPS remodeling supporting information.
(PDF)
