# Supplementary material for: Helicobacter pylori versus the Host: Remodeling of the Bacterial Outer Membrane Is Required for Survival in the Gastric Mucosa
Source: PLoS Pathog. 2011 Dec 22;7(12):e1002454. doi: 10.1371/journal.ppat.1002454 (PMC3245313; doi:10.1371/journal.ppat.1002454)
Supplement: Text S1 — H. pylori LPS remodeling supporting information. (PDF) [file ppat.1002454.s001.pdf]

Text S1

Supporting Methods

SDS-PAGE LPS Analyses. Cultures were grown to an A_{600} of ~1.0 then the equivalent to 1 ml of culture at an A_{600} of 1.0 was harvested at 16,000 x g in a microcentrifuge and washed once with PBS. Cell pellets were resuspended in 100µl of LDS sample buffer (Invitrogen) + 4% BME. Cell suspensions were boiled for 10 minutes to lyse the cells and allowed to cool. Proteinase K (New England Biolabs) was added to a concentration of 125 ng/µl and the mixture was incubated at 55°C for 16 hours. The proteinase K was heat inactivated at 100°C for 5 minutes. The proteinase K treated whole cell lysates were separated by SDS polyacrylamide gel electrophoresis using a 4-12% bis-tris gradient gel (Invitrogen). The gels were stained with Pro-Q Emerald 300 Lipopolysaccharide Gel Stain Kit (Molecular Probes).

Motility Testing. Motility was determined using semisolid brucella Broth agar supplemented with 0.4% agar and 7% FBS. Plates were inoculated using 0.5 µl of bacteria in brucella broth at A_{600} of approximately 0.05. Inoculated plates were incubated for 5 days under standard growing conditions for *H. pylori*.

Supporting Figure Legends

Figure S1. Mass spectrometry of lipid A isolated from *lpxE, lpxF,* and *lpxE/F* mutants in strain B128. Lipid A was analyzed by MALDI-TOF mass spectrometry in the negative-

ion mode. Wild type and complemented strains produced a peak at 1547.0 to 1548.0 m/z corresponding to the published wild type *H. pylori* lipid A structure. The *lpxE*, *lpxF* and *lpxE/F* mutants showed a peak at m/z 1504.6, 2090.0 and 2048.1, respectively. 1504.6 corresponds to tetra-acylated 1-phosphorylated *H. pylori* lipid A, 2090.0 to hexa-acylated 4′-phosphorylated *H. pylori* lipid A and 2048.1 to hexa-acylated *bis*-phosphorylated *H. pylori* lipid A. The lipid A structure for each strain is shown above with changes to phosphates outlined in red and changes in acylation outlined in blue.

Figure S2. Mass spectrometry of lipid A isolated from *lpxE, lpxF,* and *lpxE/F* mutants in strain X47. Lipid A was analyzed by MALDI-TOF mass spectrometry in the negative-ion mode. Wild type and complemented strains produced peaks between 1546.9 to 1548.1 m/z corresponding to the published wild type *H. pylori* lipid A structure. The *lpxE*, *lpxF* and *lpxE/F* mutants showed a peak at m/z 1504.6, 2091.1 and 2048.1, respectively. 1504.6 corresponds to tetra-acylated, 1-phosphorylated lipid A; 2091.1 to hexa-acylated, 4′-phosphorylated lipid A; and 2048.1 to hexa-acylated *bis*-phosphorylated lipid A. The lipid A structures for each strain are shown with changes to phosphate groups outlined in red and changes in acylation outlined in blue.

Figure S3. Quantitative analysis of fluorescent polymyxin B whole cell binding assay. Strains of *H. pylori* were incubated for 10 minutes in the presence of Oregon Green 514 polymyxin B (PMB-OG) at the indicated concentrations. After washing, the cells were resuspended in PBS and placed into 96-well plates for analysis. Fluorescence and A_{600} of each well was determined using a microplate reader. Each experiment was repeated

in triplicate and data reported as a ratio of fluorescence intensity to A_{600}. The *lpxE*, *lpxF* and *lpxE/F* mutants all clearly show increased binding of PMB-OG when compared to wild type. The *lpxR* and complemented strain were identical to wild type. An asterisk is used to indicate data points that are significantly different to that of wild type ($P \leq 0.002$).

Figure S4. Comparison of LPS profiles of the *lpxE, lpxF, lpxR* and *lpxE/F* mutants in strain J99. Proteinase K treated whole cell lysates from the indicated strains were separated by SDS-PAGE and stained with Pro-Q Emerald 300 Lipopolysaccharide Gel Stain Kit. The LPS profile of all strains was similar to that of wild type. LPS analysis was repeated in both B128 and X47 with similar results. LPS from *Salmonella typhimurium* strain LT2 was used as a control.

Figure S5. Comparison of motility in *lpxE, lpxF,* and *lpxE/F* mutants in strain J99. Motility was determined using semisolid brucella Broth agar supplemented with 7% FBS. Inoculated plates were incubated for 5 days under standard growing conditions for *H. pylori*. All strains showed motility similar to wild type. Motility was repeated in both B128 and X47 (data not shown) with similar results.

Table S1. Oligonucleotides

#	Primer	Sequence (5' ⇒ 3')	Restriction Site
1	Fjhp1487	GCGCGCGGATCCGACCCTAGCAGTTTGTCTTGTTTGCTC	BamH1
2	Rjhp1487	GCGCGCGAATTCTTACCATTGATAAGAAAAGCCCACCCC	EcoR1
3	Fjhp0019	GCGCGCGGATCCGCTCATCTTACACCTTGCCGGCTC	BamH1
4	Rjhp0019	GCGCGCGAATTCTTAATTAAGGTTTTTGGGGCTTGT	EcoR1
5	HP 1580-1	CGGGGTACCTTTAAGGCCATAAATCACGCC	Kpn1
6	HP 1580-2	CGCGGATCCAATTCTTGGAGAGGCATGCCAAGCGGG	BamH1

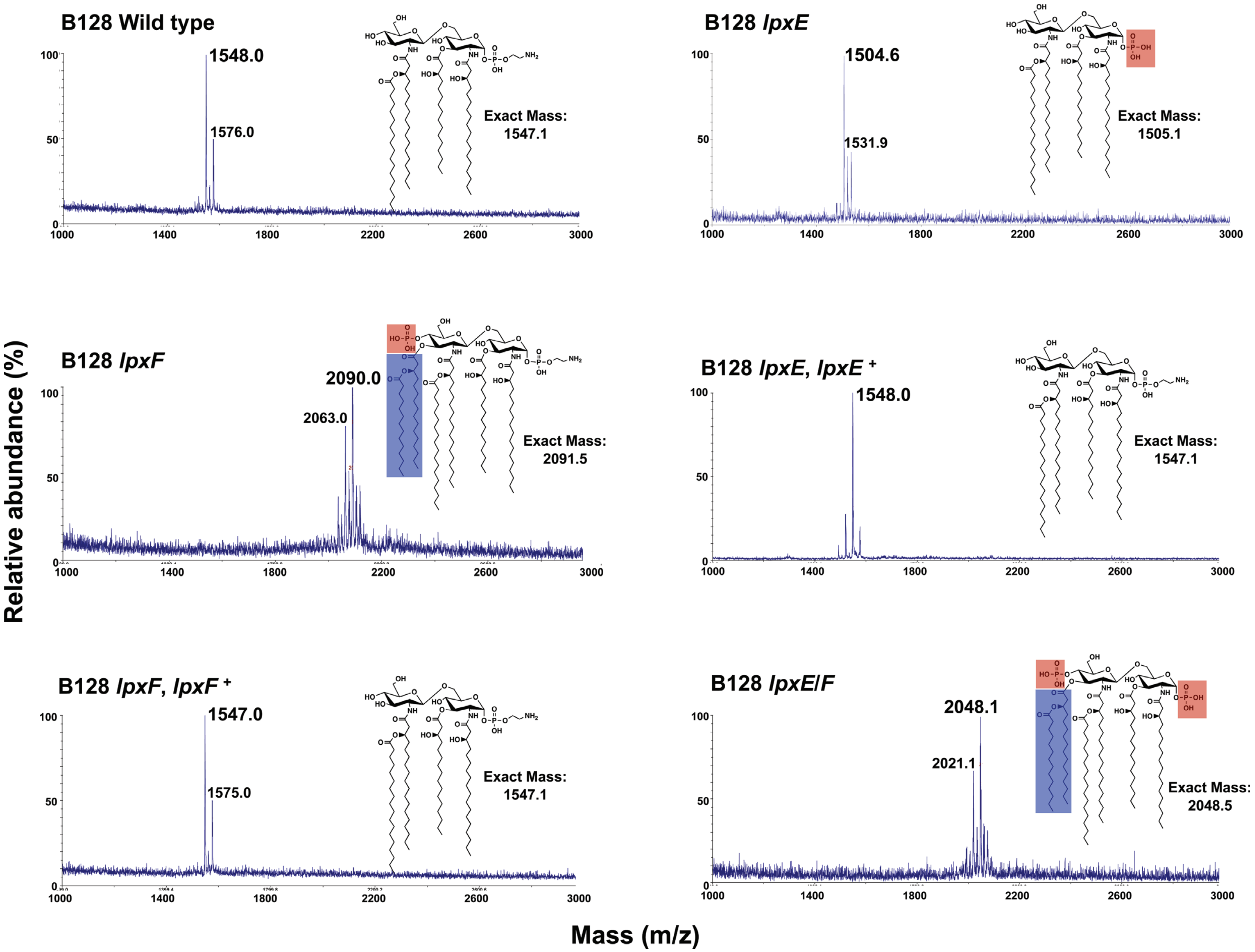
B128 Wild type
1548.0
1576.0
Exact Mass: 1547.1
B128 lpxE
1504.6
1531.9
Exact Mass: 1505.1
B128 lpxF
2090.0
2063.0
Exact Mass: 2091.5
B128 lpxE, lpxE +
1548.0
Exact Mass: 1547.1
B128 lpxF, lpxF +
1547.0
1575.0
Exact Mass: 1547.1
B128 lpxE/F
2048.1
2021.1
Exact Mass: 2048.5
Relative abundance (%)
Mass (m/z)

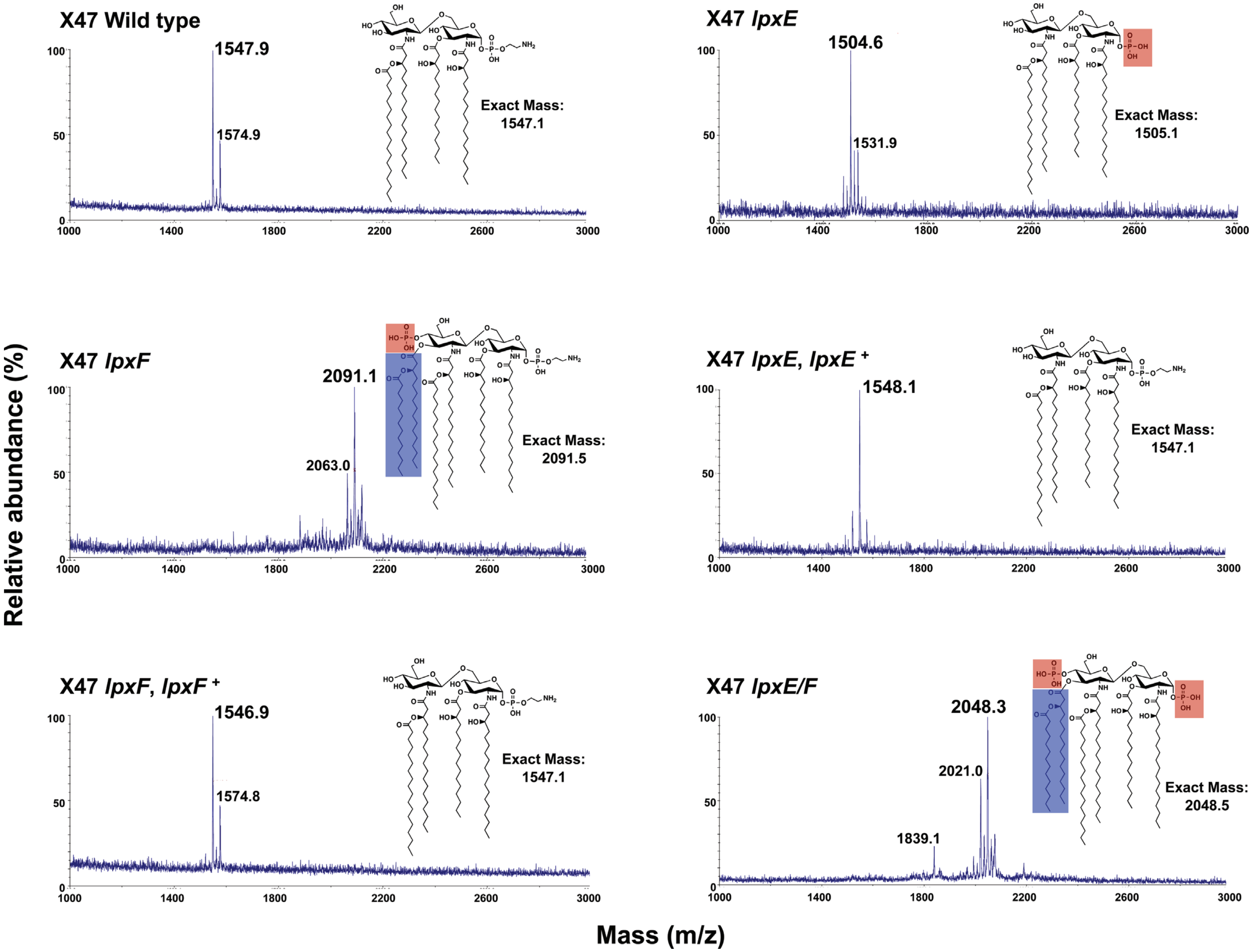

X47 Wild type
1547.9
1574.9
Exact Mass: 1547.1
X47 lpxE
1504.6
1531.9
Exact Mass: 1505.1
X47 lpxF
2091.1
2063.0
Exact Mass: 2091.5
X47 lpxE, lpxE+
1548.1
Exact Mass: 1547.1
X47 lpxF, lpxF+
1546.9
1574.8
Exact Mass: 1547.1
X47 lpxE/F
2048.3
2021.0
1839.1
Exact Mass: 2048.5
Relative abundance (%)
Mass (m/z)

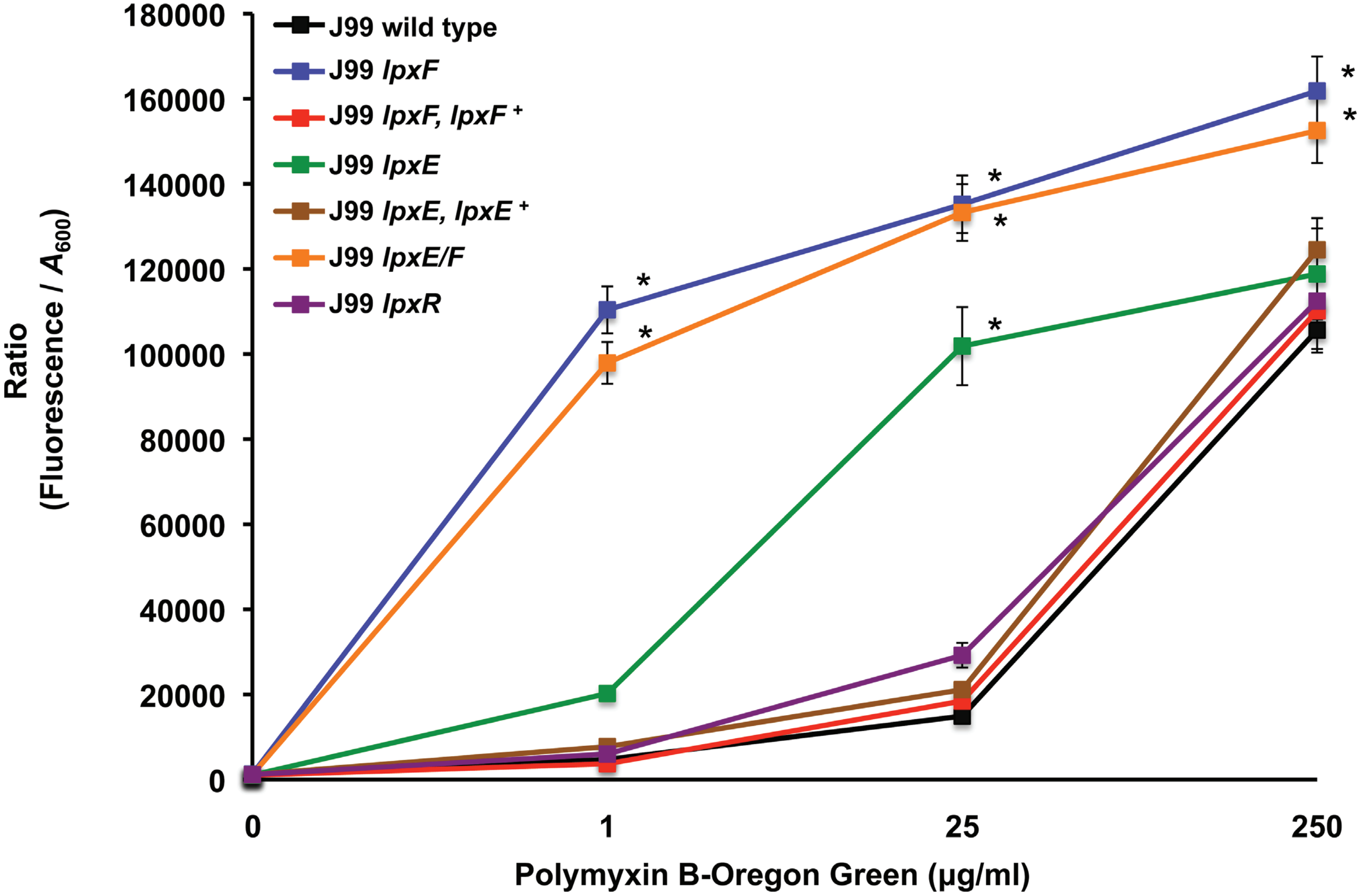

J99 wild type
J99 lpxF
J99 lpxF, lpxF +
J99 lpxE
J99 lpxE, lpxE +
J99 lpxE/F
J99 lpxR
180000
160000
140000
120000
100000
80000
60000
40000
20000
0
Ratio
(Fluorescence / A600)
0
1
25
250
Polymyxin B-Oregon Green (µg/ml)

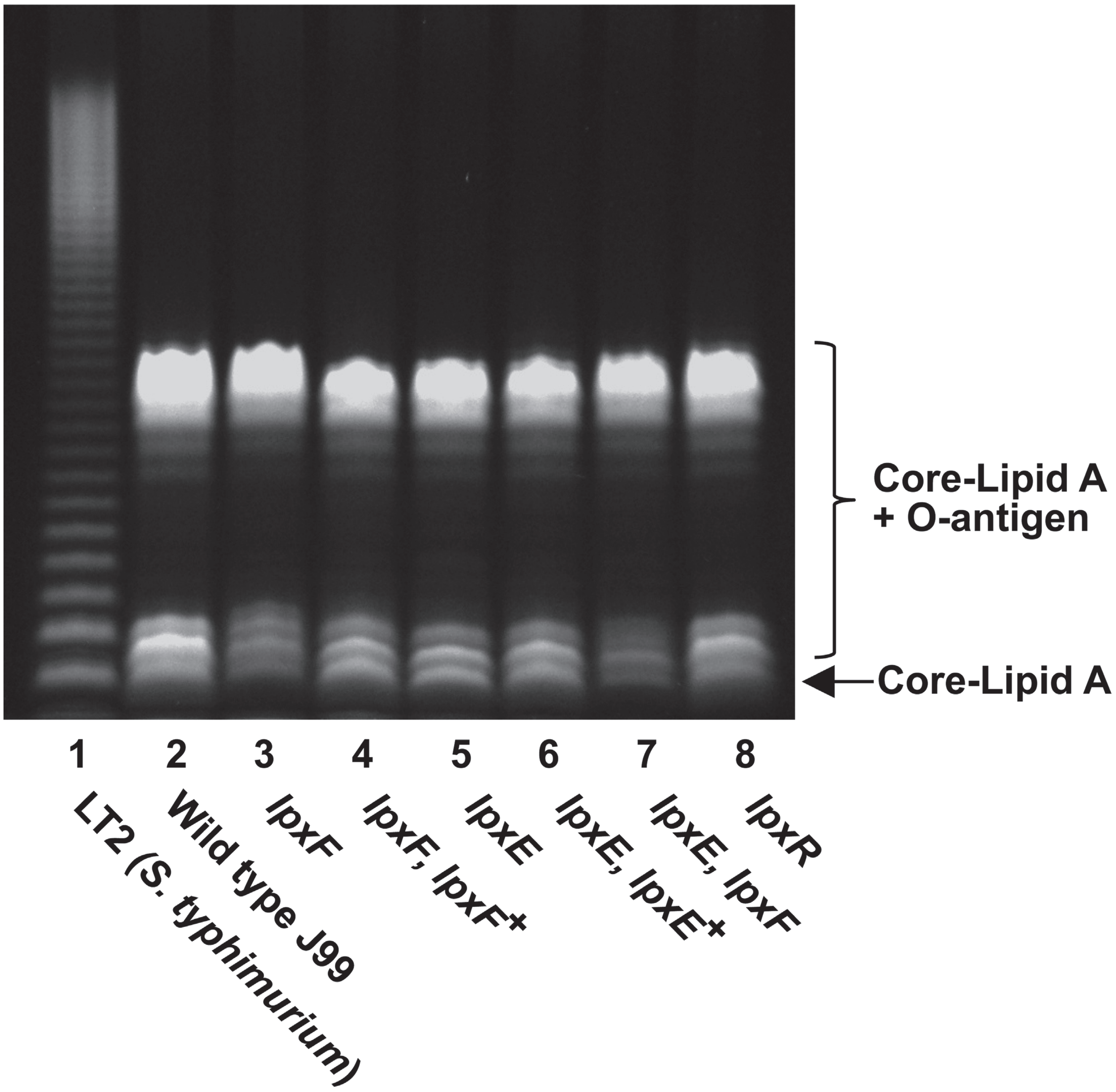
Core-Lipid A
+ O-antigen
Core-Lipid A
1
2
3
4
5
6
7
8
LT2 (S. typhimurium)
Wild type J99
lpxF
lpxF, lpxF+
lpxE
lpxE, lpxE+
lpxE, lpxF
lpxR

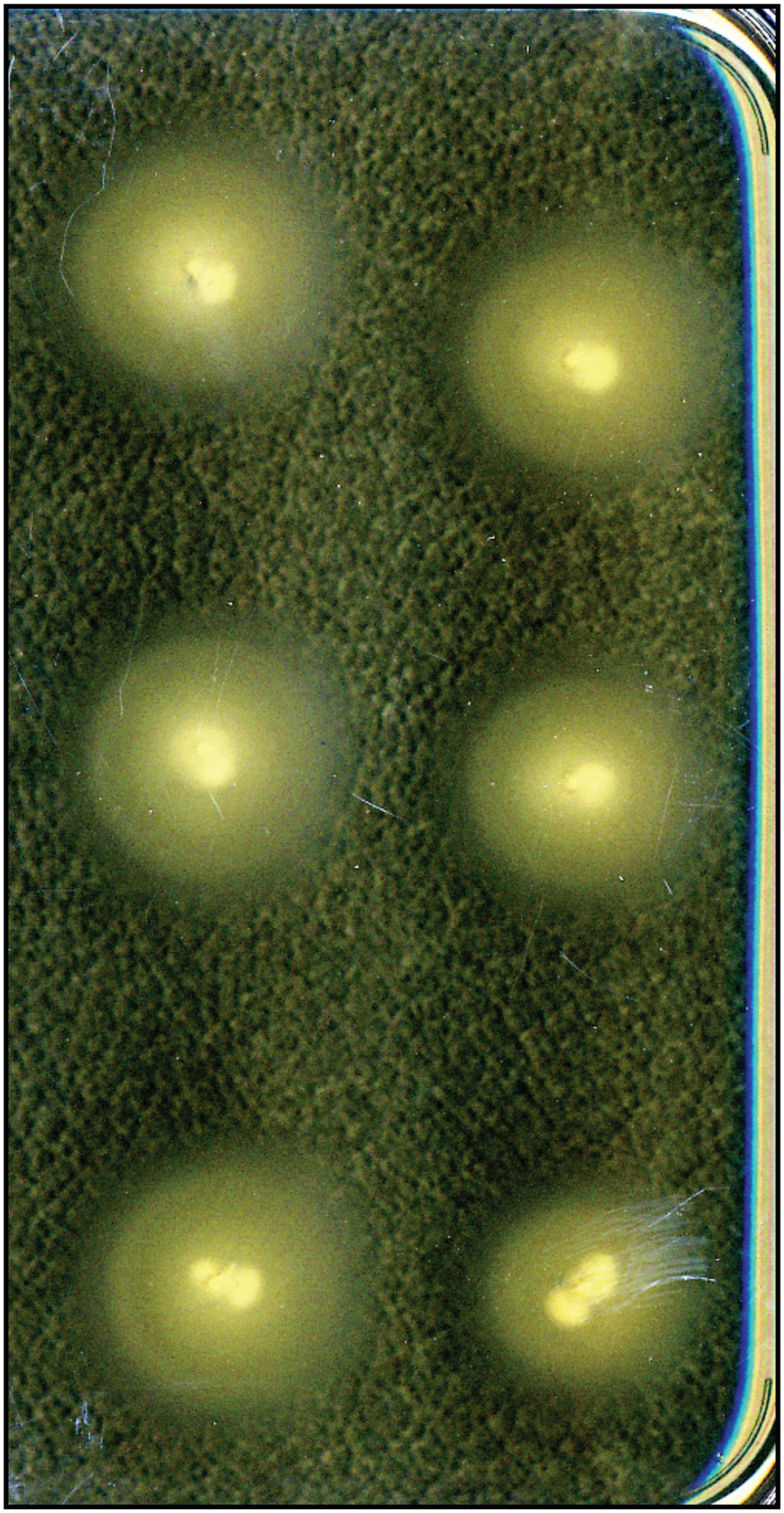
Wild type
J99
lpxE
lpxF
lpxE, lpxE +
lpxF, lpxF +
lpxE/F
